# Design of Fluorine‐Free Weakly Coordinating Electrolyte Solvents with Enhanced Oxidative Stability

**DOI:** 10.1002/anie.202506826

**Published:** 2025-07-20

**Authors:** Lennart Wichmann, Adil Aboobacker, Steffen Heuvel, Felix Pfeiffer, Robert‐Tobias Hinz, Frank Glorius, Isidora Cekic‐Laskovic, Diddo Diddens, Martin Winter, Gunther Brunklaus

**Affiliations:** ^1^ Forschungszentrum Jülich GmbH Helmholtz–Institute Münster IMD‐4, Corrensstr. 48 48149 Münster Germany; ^2^ International Graduate School for Battery Chemistry, Characterization, Analysis, Recycling and Application (BACCARA) University of Münster Corrensstr. 40 48149 Münster Germany; ^3^ Institute of Organic Chemistry University of Münster Corrensstr. 36 48149 Münster Germany; ^4^ MEET Battery Research Centre Institute of Physical Chemistry University of Münster Corrensstr. 46 48149 Münster Germany

**Keywords:** Electrochemistry, Fluorine‐free electrolyte solvents, Ion pairs, Molecular design, Weakly solvating electrolyte

## Abstract

High concentrations of conducting salt in electrolyte formulations enhance the agglomeration of ionic species, which has been demonstrated to yield anion‐derived electrode–electrolyte interphases and improved reversibility in several battery configurations. However, industrial application of these electrolytes may be limited due to high costs of electrolyte conducting salts. Here, weakly solvating electrolyte solvents with tailored coordination strength have been established as an approach to achieve ionic agglomeration at moderate conducting salt concentrations and without per‐fluorinated diluents. However, the inevitable presence of uncoordinated solvent molecules in this electrolyte concept renders them susceptible to oxidative decomposition. Although previous efforts demonstrated fluorination as an effective design strategy to tailor the oxidative stability of weakly solvating electrolytes, the per‐fluorinated solvents are toxic and harmful to the environment. Herein, the incorporation of silicon is evaluated as an eco‐friendly approach to dispel electron density of the oxygen lone pair. Though steric demand of substituents is already sufficient to tailor the coordination strength, negative hyperconjugation effectively expands the oxidative stability limit of weakly solvating electrolytes. Combining ion agglomeration and intrinsic oxidative stability, the herein introduced weakly solvating electrolyte enables a notable improvement of reversibility under eco‐friendly conditions, presenting a valid alternative to fluorinated electrolyte solvents.

## Graphical abstract

Incorporation of silicon is systematically explored as a sustainable strategy to design weakly solvating electrolytes for lithium‐based batteries. After identifying a promising molecular structure via computational screening, coordination spheres and their correlation with electrochemical key performance indicators are compared between electrolyte formulations, revealing that negative hyperconjugation enhances their oxidative stability.

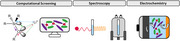

## Introduction

While increasing the potential difference between the positive and negative electrode may boost the available energy density of the respective battery cells,^[^ ^1^, ^2^, ^3^, ^4^ ^]^ it also enhances the electrochemical reactivity of electrodes, limiting their safety and reversibility.^[^ ^4^, ^5^, ^6^ ^]^ Ideally, decomposition reactions of the electrolyte yield electrode–electrolyte interphases that passivate the reactive electrodes, preventing further non‐Faradaic reactions. Thus, fine‐tuning of the electrolyte composition is essential to enable high‐energy‐density batteries with improved stability and endurance.^[^ ^7^, ^8^ ^]^ For several alkali‐ion‐ and alkali‐metal‐based battery cell chemistries, the introduction of high‐concentration electrolytes (HCEs) improved the passivation and ionic transport of the interphase,^[^ ^9^, ^10^, ^11^, ^12^, ^13^, ^14^, ^15^, ^16^, ^17^ ^]^ correlating with the formation of more homogeneous interphases that are comprised of predominantly inorganic (e.g., lithium‐fluoride or ‐oxide) instead of organic (polymeric) species.^[^ ^16^, ^18^, ^19^ ^]^ Due to the high conducting salt concentration, anions participate in the cation solvation sheath, eventually resulting in contact‐ion‐pairs (CIPs) or agglomerates (AGGs) of several ions.^[^ ^15^, ^20^ ^]^ Despite the in principle higher affinity of the hard Lewis acidic cations to the solvent molecules as hard Lewis bases, anions are forced to coordinate cations due to the scarcity of solvent molecules in typical high‐concentration electrolyte formulations.^[^ ^21^ ^]^ Decomposition of these anion‐rich coordination environments yields interphases that are comparatively rich in inorganic domains, bestowing superior electrochemical performance on corresponding cells.^[^ ^22^, ^23^ ^]^ To reduce conducting salt concentration and further enhance the ion agglomeration, HCEs are often diluted by a non‐coordinating co‐solvent, yielding localized high‐concentration electrolytes (LHCEs). Maintaining the conducting salt to solvent ratio in the active coordination sphere while separating these spheres with diluent molecules, similar electrochemical cell performance is achieved at lower conducting salt concentrations and viscosities.^[^ ^15^, ^24^ ^]^ In these electrolyte formulations, solvents act as surfactants at interfaces between salt agglomerates and diluents, yielding micelle‐like structures with higher ion agglomeration on their inside.^[^ ^20^ ^]^

Despite the improved longevity when employing HCEs and LHCEs in several cell chemistries,^[^ ^9^, ^10^, ^11^, ^12^, ^13^, ^14^, ^15^, ^16^, ^17^ ^]^ potential commercialization is considered unlikely. Especially certain diluents, which are the major constituent of most LHCEs, require costly synthesis and are on the verge of being banned inside the European Union along with other per‐fluorinated alkyl substances (PFAS) due to their persistence, carcinogenicity, and likely negative environmental impact.^[^ ^25^, ^26^, ^27^ ^]^ Solely invoking HCEs without the addition of diluents also does not present a feasible approach for commercialization, since the conducting salt concentrations of up to 10 mol L^−1[^ ^16^ ^]^ raise concerns in view of their unfavorably high costs.^[^ ^24^ ^]^ Thus, strategies to enhance anionic participation in the respective solvation sheath of lithium ions at moderate conducting salt concentrations and without the presence of fluorinated co‐solvents are necessary. In this respect, tailoring the Lewis basicity or coordination strength of the considered solvents denotes a highly promising and practical approach, a concept referred to as weakly solvating electrolytes (WSEs). While weaker ion coordination increases the involvement of anions in the solvation sheaths of cations, sufficient solvation strength is still a prerequisite to allow for conducting salt dissolution and ionic transport.^[^ ^28^ ^]^ Recent works achieved this by increasing the solvents steric demands or by fluorine substitution, limiting ion coordination either via increased distances to the cation or reduced electron density at the solvents oxygen moieties due to inductive effects.^[^ ^28^, ^29^, ^30^, ^31^, ^32^ ^]^ Though particularly the latter approach resulted in electrolytes that boost the reversibility of lithium metal batteries, structural similarity of designed solvent molecules with previously discussed diluents also renders them non‐eco‐friendly and affected by the envisaged ban on PFAS.^[^ ^25^, ^26^ ^]^ While a tailored steric demand of an electrolyte solvent is suitable to limit its coordination strength,^[^ ^33^, ^34^ ^]^ the free lone pair of non‐coordinating ether moieties is susceptible to oxidative decomposition.^[^ ^21^, ^22^ ^]^ Thus, we herein explore negative hyperconjugation as a sustainable approach to design weakly solvating electrolytes. One example of negative hyperconjugation includes direct vicinity of silicon atoms to a hydroxyl‐substituted carbon center, where the antibonding Si─C orbital (σ*_Si─C_) has an energy level comparable to the oxygen p orbital (p_O_).^[^ ^35^ ^]^ This enables delocalization of electron density from the lone electron pair of the oxygen moiety, thereby reducing the solvent's coordination strength.^[^ ^35^, ^36^, ^37^ ^]^ Furthermore, less electron density in the lone pair orbital may also enhance the solvent's intrinsic oxidative stability. Despite the introduction of siloxane‐based electrolyte formulations,^[^ ^38^, ^39^, ^40^ ^]^ fundamental understanding of the impact of silicon substitution on the design of novel electrolytes has yet to be obtained. Since in previous reports, a lack of structural comparability between the considered electrolyte solvents convoluted insights on silicon incorporation with steric effects, our focus lies on providing a systematic and fair comparison of electrolytes containing the structurally similar solvents methyl *tert*‐butyl ether (MTBE) and methoxytrimethylsilane (MTMS), each at various conducting salt‐to‐solvent ratios. Following a computational screening by means of gain in free energy from solvent‐lithium ion coordination, the actual impact of silicon substitutions on the corresponding lithium ion coordination environments is determined, combining data from molecular dynamic (MD) simulations and Raman and NMR spectroscopy, respectively. Also, the considered electrolyte formulations are compared to benchmark electrolytes in view of battery‐related key performance indicators including the ionic conductivity, electrochemical stability window and achievable reversibility of lithium inventory.

## Results and Discussion

### Computational Screening

**Figure 1:**
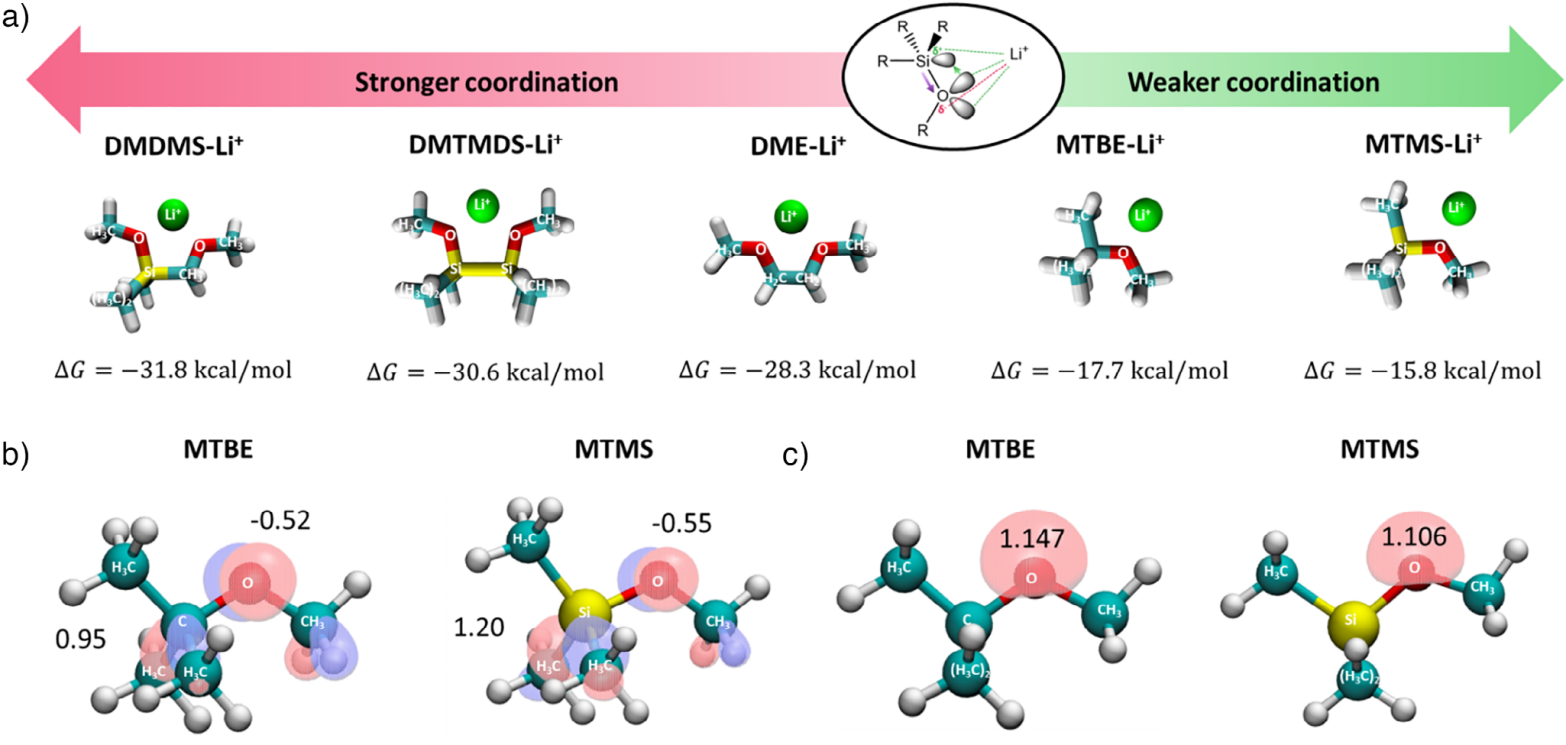
Computational screening of potential electrolyte solvents: a) Gain in free‐energy ***Δ**G* from the coordination of the respective solvent with a “free” lithium ion. b) Highest occupied molecular orbitals (HOMO) of MTBE and MTMS and atomic charges on O and Si/C determined via DFT calculations and charges from electrostatic potentials exploiting a grid‐based method (CHELPG),^[^ ^44^ ^]^ respectively. c) Electron population in the lone‐pair orbital of oxygen for MTBE and MTMS determined using Pipek–Mezey^[^ ^45^ ^]^ localization.

To screen for suitable solvent molecules in which negative hyperconjugation between oxygen lone‐pair electrons and the anti‐bonding Si─C orbital decreases the coordination strength, density functional theory (DFT) calculations of the binding energy between the solvents and a “free” lithium cation were carried out. While this approach neglects steric effects experienced upon cation coordination with several ligands, the gain in free energy Δ*G* from geometry‐optimized solvent–Li^+^ coordination may be employed as an indicator for the solvent's Lewis basicity.^[^ ^28^, ^34^, ^41^ ^]^ With a typical root‐mean‐square deviation of 0.7 kcal mol^−1^, the selected DFT functional offers a reliable approach to identify solvents with a reduced coordination strength due to incorporation of silicon.^[^ ^42^ ^]^ As many localized high‐concentration electrolytes (LHCEs) employ 1,2‐dimethoxyethane (DME) as electrolyte solvent,^[^ ^15^, ^30^, ^43^ ^]^ it is herein employed as a reference solvent and starting point for computational screening. However, implementing one or two silicon moieties in the center of DME increases the gain in free energy Δ*G* from coordination to Li^+^ (Figure 1a), indicating enhanced rather than decreased coordination strength.

When incorporating silicon into monodentate methyl *tert*‐butyl ether (MTBE), DFT calculations predict a lower gain in free energy Δ*G*, indicative of reduced coordination strength. Despite limited comparability between mono‐ and bidentate solvents, differences in Δ*G* when substituting carbon with silicon in DME and MTBE suggest a complex interplay of phenomena involved. Besides negative hyperconjugation, steric and electronic effects due to the increased size and reduced electronegativity of silicon instead of carbon also have to be considered. While a more polarized Si─O bond might intuitively be anticipated to increase the coordination strength of oxygen moieties, previous computational studies discussed electrostatic repulsion between the cation and positively polarized Si moieties as a counteracting effect.^[^ ^46^ ^]^ Thus, molecular structure and geometric arrangement of the coordinating complex also impact the binding strength. Next to the electronic structure of the electrolyte solvent, the bonding character toward the cation also needs to be considered. While a more dipolar bonding character increases the impact of bond polarization, coordination in the form of charge‐transfer donation is expected to depend on the oxygen lone pair population.^[^ ^46^ ^]^ When replacing carbon with silicon atoms, higher bond polarization may enhance the dipolar coordination strength, whereas negative hyperconjugation is expected to lower the charge‐transfer coordination strength.

The interplay of these steric and electronic effects helps to understand the contrary trends in free‐energy Δ*G* (Figure 1a) for silicon substitution with different solvent molecules. Comparing atomic charges in MTBE and the silicon‐substituted analogue methoxytrimethylsilane (MTMS, Figure 1b), the Si─O bond is more polarized than the C─O bond, yielding an increased electron density at the oxygen moiety in MTMS. However, compared to the decrease of partial atomic charge on silicon (−0.25), the increase of atomic charge on oxygen (+0.02) is notably lower. This can be rationalized when separately evaluating the oxygen lone‐pair orbital population (Figure 1c), which decreases due to negative hyperconjugation. Since negative hyperconjugation counteracts the gain in electron density from increased bond polarization, the atomic charge of oxygen changes less upon silicon substitution than that of its bonding partner. Furthermore, the bond angle of oxygen increases from 117.5 degrees in MTBE to 122.7 degrees in MTMS, which can be attributed to delocalization of oxygen lone pair electron density. Natural bond orbital analysis of the antibonding Si─C orbital (σ*_Si─C_) receiving electron density via negative hyperconjugation corroborates the findings, since electron population is increased by 65% compared to the antibonding C─C orbital (σ*_C─C_) (Table S4).

With the gain in free energy Δ*G* for coordination of lithium with MTMS being lower, the reduced lone‐pair orbital population apparently has a greater impact on the ion coordination strength than the total atomic charge of oxygen in this case. For bidentate DME‐based solvents, DFT calculations suggest an increase rather than a decrease in coordination strength when incorporating silicon (Figure 1a). Since increased atomic charges of oxygen (Figure S4a) as well as depopulation of the oxygen lone‐pair orbitals (Figure S4b) are also observed with DME analogues, we consider differences in the molecular arrangement of DME‐ and MTBE‐based solvents the explanation for a gain instead of a decrease in free energy Δ*G*. Evaluating the geometry‐optimized molecular structures, the distance of bridging atoms between oxygen moieties increases from 1.50 Å (C─C) to 1.94 Å (C─Si) and 2.35 Å (Si─Si) upon consecutive substitution of C with Si. Since the conformation of Si‐substituted DME analogues coordinating to lithium experiences less strain at larger distance from the oxygen moieties, coordination becomes more favorable, and the gain in free energy Δ*G* is further enhanced.

With MTBE and MTMS being of similar steric demand but expected to display different coordination strength judging from DFT calculations, these solvents are suitable model compounds to explore and understand silicon substitution as a potential design strategy to achieve eco‐friendly, weakly coordinating electrolyte solvents. In addition, DME will be evaluated as a benchmark electrolyte‐solvent since it is predominantly utilized as the electrolyte solvent in ether‐based LHCEs. Since superior electrochemical performance with such electrolytes in high‐voltage lithium‐metal batteries^[^ ^15^, ^16^, ^47^, ^48^ ^]^ was demonstrated to originate from anion‐derived interphases that are created by high anionic participation in the coordination of Li^+^,^[^ ^20^, ^23^, ^49^ ^]^ molar conducting salt to solvent ratios are commonly used to specify electrolyte formulations rather than referring to the conducting salt concentration.^[^ ^15^, ^50^ ^]^ To enable a reasonable comparison of bidentate reference solvent DME and monodentate solvents MTBE and MTMS, a molar conducting salt to oxygen moiety ratio is herein established, doubling the molar ratio of solvent when substituting DME with MTBE or MTMS (Figure S1). Here, the molecular ratio of 1.0:1.2 for LiFSI and DME in previously reported high‐concentrated electrolytes (HCEs) is used as a reference point.^[^ ^15^ ^]^ Electrolyte formulations with lower conducting salt‐to‐solvent ratios will be referred to as “moderate‐concentration electrolytes” (MCEs) and “low‐concentration electrolytes” (LCEs), respectively. It should be noted that doubling the molar ratio of solvent when substituting DME with MTBE or MTMS results in varying conducting salt concentration and thus physiochemical properties such as viscosity. However, since the amount of conducting salt and available oxygen moieties for its dissociation are decisive for achieving exceptional electrochemical performance of HCEs and LHCEs,^[^ ^15^, ^20^ ^]^ comparability in this regard is prioritized against other physiochemical properties.

### Characterization of Coordination Spheres

**Figure 2:**
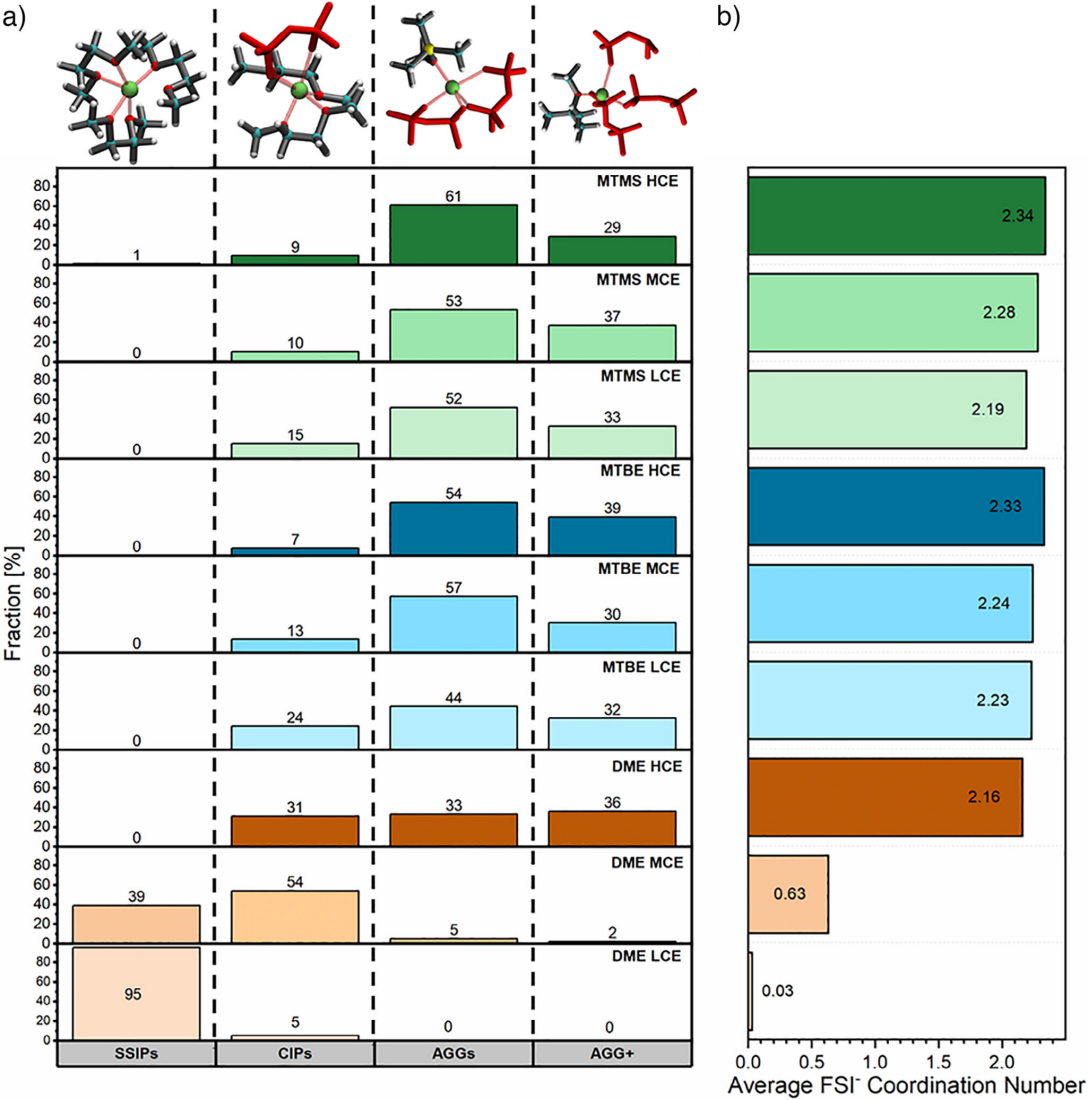
Coordination environments analyzed via MD simulations: a) Distribution of Li^+^ and FSI^−^ ion pairings evaluated on a frame at the end of MD run. b) Average coordination number of FSI^−^ to Li^+^ over multiple frames (cut‐off distance of 5 Å between Li and N was determined from the RDFs).

With the salt‐to‐solvent ratios specified (Figure S1), molecular dynamic (MD) simulations allow computational evaluation of the electrolyte model structures with varying electrolyte solvents and conducting salt‐to‐solvent ratios, including steric effects and complex geometries. Extracting the radial distribution of anions around lithium cations (Figure S5), ion clustering can be expressed as a distribution of coordination numbers between Li^+^ and FSI^−^ ions (Figure 2). Here, the lithium coordination spheres are categorized in the form of solvent‐separated ion pairs (SSIPs, Li(solvent)*_n_*), contact ion pairs (CIPs, Li(FSI)_1_(solvent)*_n_*_−1_), ion agglomerates (AGGs, Li(FSI)_2_(solvent)*_n_*_−2_), and extensive ion agglomerates (AGG+, Li(FSI)_≥3_(solvent)*_n−_*_≥3_).^[^ ^20^, ^51^ ^]^ A detailed distribution of anion coordination numbers further distinguishing AGG+ species is depicted in Figure S6. Since the MTMS‐based LHCE yielded an abnormally high amount of ion clustering in our MD simulations (Figure S7), most likely related to the parameters requiring different scaling of charges, solely data of binary LiFSI‐solvent systems (LCEs, MCEs, and HCEs) are discussed.

While all the considered electrolytes display ion agglomeration at high salt‐to‐solvent ratios, substantial differences between DME‐ and MTBE‐ or MTMS‐based electrolytes become evident in MCE and LCE formulations. With DME as the electrolyte solvent, extensive ion agglomeration is exclusively observed in the HCE. Due to DMEs comparatively high coordinating strength, solvent‐separated ion pairs are predominantly observed in the LCE. Here, anions are only involved in the coordination sheath when limited amounts of solvent molecules are available. In contrast, the MTBE‐ and MTMS‐based electrolytes exhibit an average anion coordination number >2 at all conducting salt‐to‐solvent ratios. This agglomeration of ions independent of the salt‐to‐solvent ratio is characteristic of weakly coordinating solvents. Nevertheless, the involvement of FSI^−^ in the Li^+^ coordination spheres also slightly increases with the salt‐to‐solvent ratio. Since this behavior is already observed for MTBE‐based electrolytes, the sterically demanding *tert*‐butyl and trimethylsilyl substituents appear to already limit interactions between the oxygen moieties and lithium ions. Comparing MTBE and MTMS electrolytes, the further reduced coordination strength with MTMS, anticipated from the lower binding strength in the DFT calculations, only results in a minor increase in anionic coordination numbers at medium and high conducting salt concentrations. In the case of weakly solvating LCEs, MTBE even exhibits higher average FSI^−^ coordination numbers (2.23 versus 2.19).

While the first coordination sphere of lithium ions impacts the electrolyte's electrochemical stability and thus interphase formation and composition,^[^ ^16^, ^52^ ^]^ the global structure is crucial for the ionic conductivity in highly aggregated electrolytes.^[^ ^50^, ^52^, ^53^, ^54^ ^]^ Instead of vehicular lithium transport in conjunction with its solvation shell observed in conventional electrolytes, lithium ion diffusion at high ion aggregation predominantly proceeds through ion hopping among different coordination sites,^[^ ^54^ ^]^ similar to ionic transport in a crystal lattice. With increasing “structural” transport, factors such as the electrolyte viscosity and ion mobility become less relevant.^[^ ^50^ ^]^ Since the proximity of coordination spheres determines the distance a lithium ion can migrate via hopping,^[^ ^53^, ^54^ ^]^ interconnection of coordinating sites based on a percolating network facilitates ion transport in electrolytes with high degrees of ion clustering.^[^ ^54^ ^]^ Analysis of Li─Li radial distribution functions (RDFs, Figure S8) enables a perspective beyond the first coordination shell, providing valuable insights on the size of the ionic clusters. Here, a distance below 7 Å is identified as a distinct coordination environment with the lowest Li─Li distance.

**Figure 3:**
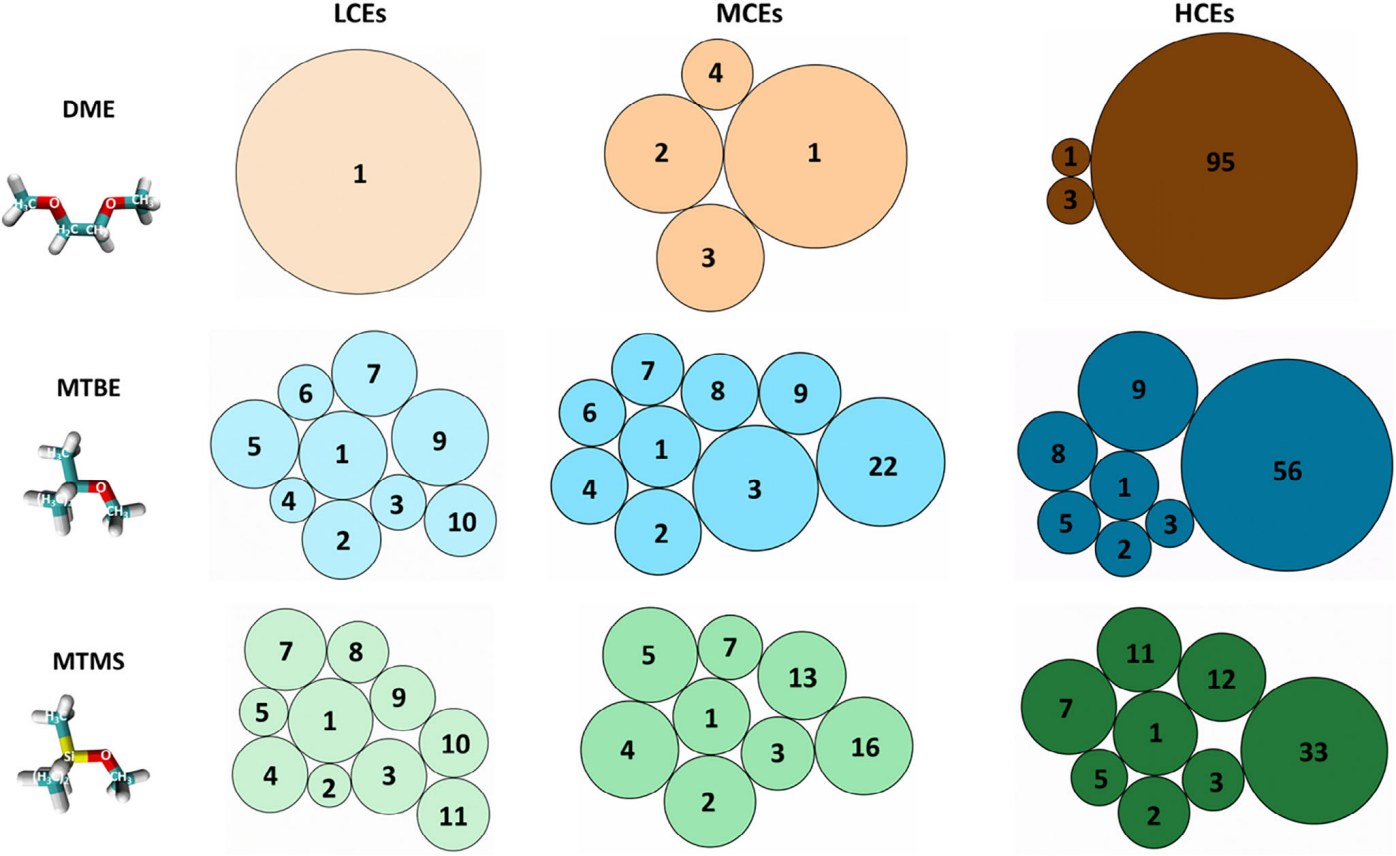
Graphic representation of Li─Li cluster sizes and their population derived from cluster analysis of a frame at the end of the MD production run. Since distances below 7 Å are identified as the minimal distinct environment in Li─Li RDFs, lithium atoms below this cut‐off are considered to be in the same cluster. Numbers in circles represent the cluster size, while the circle area indicates its relative population.

Since lithium ions sharing the same anion in their respective coordination sphere are expected to display the lowest Li─Li distance and are essential to form a percolating network,^[^ ^54^ ^]^ 7 Å is selected as cut‐off criterion for interconnected lithium ions that facilitate ion hopping. By analyzing the Li─Li RDFs with respect to this distance, the distribution of lithium cluster sizes is determined (Figure 3). While a prevalence of solvent coordination results in the absence of ion clusters and therefore a cluster size of one in DME‐based LCE, high degrees of anion coordination yield a percolating ionic network in the HCE, involving 95 of 100 lithium ions in the MD simulation box. In the DME‐based MCE, ion agglomerates can be found, but contact‐ion pairs are the most probable coordination environment (Figure 2). Thus, ionic clusters containing more than one lithium ion occur but are less prominent and limited in size.

For the weakly solvating MTBE‐ and MTMS‐based electrolytes, FSI^−^ coordination numbers of two are the most probable coordination environment at all salt‐to‐solvent ratios (AGGs, Figure 2). With lithium coordination environments containing three or even more anions being the second most common cluster configuration, interconnection of ion agglomerates and hence a variety of cluster sizes is observed in all weakly solvating electrolytes (Figure 3). While the cluster size also increases with the salt‐to‐solvent ratio in the weakly solvating electrolytes, the differences between LCEs, MCEs, and HCEs are not as pronounced as in the DME‐based electrolytes. Instead of the first coordination sphere, which limits the size of ion agglomerates in DME‐based LCE and MCE, the salt‐to‐solvent ratio itself is the limiting factor in the case of weakly solvating electrolytes. While the abundance of ionic species at high salt‐to‐solvent ratios forces their proximity and merges them into larger clusters, less ionic species are present at low salt‐to‐solvent ratios and are thus separated by excessive weakly coordinating solvent molecules. Comparing the HCEs based on different electrolyte solvents, a decrease in coordination strength correlates with smaller cluster size. Here, a more uniform distribution of contact ion pairs, ion agglomerates, and extensive ion agglomerates, as observed in DME‐based HCE (Figure 2), appears to be favorable to form larger ionic clusters.

**Figure 4:**
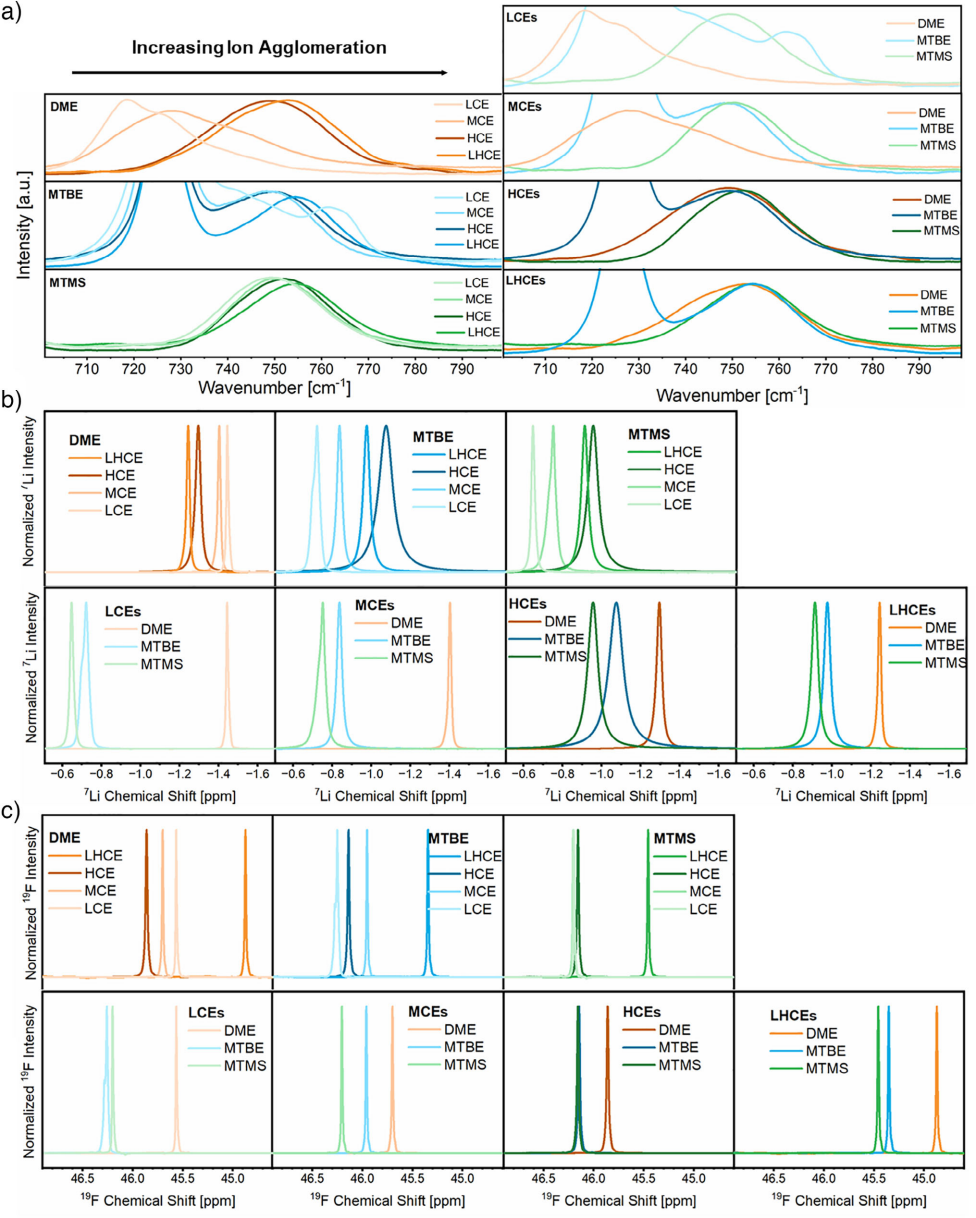
Spectroscopic characterization of all considered electrolyte formulations: a) Comparison of FSI^−^ Raman bands for identical electrolyte solvents at varying salt‐to‐solvent ratios (left) and varying solvents at identical salt‐to‐solvent ratios (right). All the spectra are scaled to display comparable intensities. b) Comparison of normalized ^7^Li NMR spectra for identical solvents at varying salt‐to‐solvent ratios (top) and varying solvents at identical salt‐to‐solvent ratios (bottom). c) Comparison of normalized ^19^F NMR spectra for identical solvents at varying salt‐to‐solvent ratios (top) and varying solvents at identical salt‐to‐solvent ratios (bottom).

To complement the computational characterization of lithium coordination environments experimentally, Raman spectroscopy is a suitable technique (Figure 4a). Particularly the S─N─S bond symmetric stretching vibrational mode in FSI^−^ affords insights into the actual involvement of anions in the lithium coordination shell, since solvent‐separated ion pairs, contact ion pairs, and ion agglomerates exhibit an increasing shift toward higher wavenumbers.^[^ ^9^, ^20^, ^29^ ^]^ In good agreement with the MD data, DME‐based electrolytes exhibited a concentration dependence for the extent of ion clustering, with predominantly solvent‐separated ion pairs (∼720 cm^−1^)^[^ ^20^ ^]^ at low and ion agglomerates (745–755 cm^−1^)^[^ ^20^ ^]^ at high conducting salt to solvent ratios. Adding a fluorinated diluent, the clustering of ions is further enhanced, attributed to a phase separation between diluent and the ionic clusters. Since this requires the solvent to act as a surfactant, its availability for lithium coordination inside ionic clusters is further decreased, which boosts participation of anions in lithium ion coordination environments.^[^ ^20^ ^]^ The analysis of the O─C─C─O vibrational band of DME, which shifts to higher wave numbers upon coordination to Li^+^,^[^ ^20^, ^34^ ^]^ supports the correlation between the extent of ion agglomeration and the availability of remaining non‐coordinating DME molecules. Scaling of all Raman spectra for the DME‐based electrolytes to have the same intensity at the band associated with Li^+^‐coordinated DME (Figure S9), a decay in non‐coordinating DME molecules with increasing conducting salt concentration is clearly observable.^[^ ^20^, ^51^, ^55^ ^]^ With MTBE‐ and MTMS‐based electrolytes, Raman spectra display the formation of ion agglomerates at all salt‐to‐solvent ratios, as predicted in MD simulations. While increasing the conducting salt concentration as well as addition of the diluent facilitates ion agglomeration slightly, the impact is less pronounced compared to DME‐based electrolytes (Figure 4a).

MTMS‐based electrolytes exhibit the least concentration‐dependent variation in Raman shift of the FSI^−^ band. Compared to MTBE‐based electrolytes, ion agglomeration is slightly increased for the MCE, HCE, and LHCE, respectively. At low salt‐to‐solvent ratio, however, a shoulder peak at higher FSI^−^ (Figure 4a) and lower oxygen (Figure S9) wavenumbers (i.e. the regions of ion agglomerates) is observed for the MTBE‐based LCE. Except for this shoulder peak suggesting an additional coordination environment with enhanced ion agglomeration, the trends determined from Raman spectroscopy confirm the average FSI^−^ coordination numbers extracted from MD simulations. However, it should be noted that the solvent bands, namely the symmetric C─CH_3_ stretching band at 722 cm^−1^ for MTBE^[^ ^56^ ^]^ and a weak Si─CH_3_ stretching band at 750 cm^−1^ for MTMS,^[^ ^57^ ^]^ interfere in the wavelength range of FSI^−^ bands and hence may impact spectral deconvolution.

**Figure 5:**
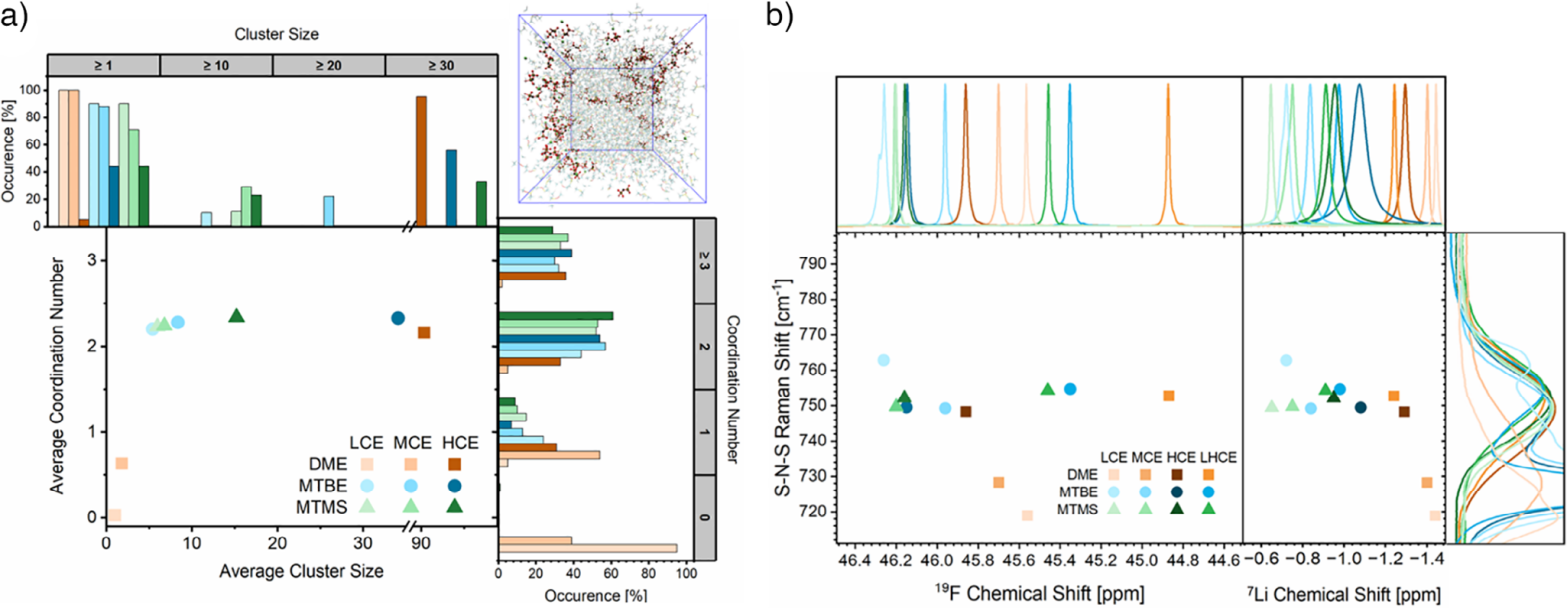
Summary of the (a) computational and (b) experimental results on the structure of considered electrolytes.

Next to Raman spectroscopy, the ^7^Li and ^19^F chemical shifts of the electrolyte constituents determined by nuclear magnetic resonance (NMR) spectroscopy are also often evaluated to assess the extent of ion clustering present within electrolytes. Here, the electron density present at probed nuclei induces a local magnetic field, which shields them from the external magnetic field.^[^ ^58^ ^]^ Thus, nuclei with high electron density in their vicinity display lower NMR chemical shifts, while nuclei with neighboring electronegative atoms that are withdrawing electron density appear at higher chemical shifts.^[^ ^58^ ^]^ When considering 3D structures such as Li^+^ coordination environments, contribution of local electron density in the vicinity of a probed nucleus is orientation‐dependent and rather complex. Furthermore, it is unclear whether coordination of Li^+^ by FSI^−^ or the solvent molecules results in a stronger shielding and increase of electron density at the Li^+^ nucleus. In recent literature, both higher^[^ ^34^, ^41^ ^]^ and lower^[^ ^22^, ^28^, ^29^, ^33^ ^]^ chemical shifts of ^7^Li nuclei were interpreted to be indicative of increased anion participation within the lithium solvation sheath. In our case, electrolytes with reference solvent DME exhibit a clear trend of more positive ^7^Li chemical shifts with increasing salt‐to‐solvent ratios and thus ion agglomeration (Figure 4b). Addition of the diluent, known to enhance anion participation by forming micelle‐like structures,^[^ ^20^ ^]^ also results in increased ^7^Li NMR chemical shifts. Comparing NMR and Raman shifts (Figure 5b), we can establish a linear correlation of higher ^7^Li NMR chemical shifts and the presence of more anionic coordination environments in the case of DME‐based electrolytes. Replacing DME with the weaker solvating MTBE yields increased ^7^Li NMR chemical shifts at all considered salt‐to‐solvent ratios, also aligning with the increased ion agglomeration determined from MD and Raman data. Fittingly, substitution of MTBE with MTMS further increases the ^7^Li chemical shifts of all electrolyte compositions, except for low‐concentration electrolytes. Here, the MD and Raman data indicate enhanced ion agglomeration with MTBE, while their ^7^Li chemical shifts suggest stronger ion agglomeration with MTMS. Though Raman and NMR spectroscopy both display shoulder peaks indicative of separate coordination environments with increased ion agglomeration for the MTBE LCE, these coordination environments cannot be identified in MD simulations. Comparing ^7^Li NMR chemical shifts for MTBE‐ and MTMS‐based electrolytes at different salt‐to‐solvent ratios, the previously established approximation of higher ^7^Li chemical shifts being indicative of increased ion agglomeration would suggest stronger ion agglomeration at lower salt‐to‐solvent ratios. Since the opposite, i.e. slightly increasing anionic participation at higher salt‐to‐solvent ratios, was established via Raman and MD simulations, the simplified interpretation of ^7^Li NMR shifts apparently comes to its limits. Instead of anion participation in the first coordination sphere of lithium ions, we assign the more positive ^7^Li chemical shifts at lower conducting salt concentrations to originate from the global electrolyte structure. Here, MD simulations of weakly solvating electrolytes evidenced ionic clusters to be larger at high salt‐to‐solvent ratios and more separated by excessive weakly solvating electrolytes at low salt‐to‐solvent ratios (Figure 3). Since the lithium ions located towards the center of larger ionic clusters in HCEs experience stronger shielding from the external magnetic field than in their smaller and more isolated counterparts within the LCEs, their average ^7^Li NMR chemical shift is reduced. This is also supported by a decreased peak width at low salt‐to‐solvent ratios, where the position of lithium nuclei in ionic clusters is less diverse due to the ionic networks being smaller in size. With increasing size of the ionic cluster networks at higher salt‐to‐solvent ratios, the position of the considered lithium nucleus has higher variability, yielding a broader peak. Here, it should be noted that the phenomenon of ionic clusters being more isolated at low concentrations is specific to the weakly solvating electrolytes. For strongly coordinating electrolyte solvents such as DME, the decrease of the salt‐to‐solvent ratio also affects the first coordination shell. Thus, ionic agglomerates only exist in the form of a percolating network. Nevertheless, larger cluster sizes also introduce a higher variability in shielding of lithium nuclei, as perceptible by the difference in peak widths between DME‐based LCE and HCE. As illustrated by these observations, NMR spectroscopy also contains information on the global electrolyte structure, which deepens our understanding but is also more complex to interpret accurately. In contrast to Raman data reflecting direct coordination environments of both anions and solvents, a straightforward interpretation of higher or lower ^7^Li chemical shifts being indicative of stronger ion clustering is not feasible when characterizing weakly solvating electrolytes.

In the ^19^F NMR spectra, the peak width for FSI^−^ in weakly solvating electrolytes at different salt‐to‐solvent ratios does not vary notably (Figure 4c), indicating that cluster size effects have less impact on ^19^F NMR chemical shifts. For the MTMS‐based LCE, MCE, and HCE, ^19^F NMR chemical shifts are almost identical, aligning with only little concentration dependence of anion coordination previously derived from Raman and MD data. For MTBE‐based electrolytes, however, the observed ^19^F chemical shifts vary with the salt‐to‐solvent ratio, though Raman and MD data also only displayed minor differences in anionic Li^+^ coordination numbers. Considering that the coordination of Li^+^ eventually withdraws electron density from FSI^−^, an increase in chemical shift of FSI^−^ with more pronounced ion agglomeration is expected. Similar to the chemical shift of ^7^Li, a linear relation between ^19^F chemical shift and Raman wavenumbers can be established for the DME‐based electrolytes but not the weakly solvating electrolytes (Figure 5b). For all HCEs, the addition of the diluent reduces the chemical shift considerably. While this would suggest decreased participation of FSI^−^ in the Li^+^ solvation sheath, Raman and ^7^Li NMR spectroscopy established an increase rather than a decrease of anionic coordination going from HCEs to LHCEs. Thus, we consider this to be an effect stemming from micelle‐like structures of the LHCEs rather than from an actual decrease of ion agglomeration. Comparison of the ^19^F chemical shifts of MTBE‐ and MTMS‐based electrolytes (Figure 4c) agrees well with data from MD simulations and Raman spectra in all aspects, indicating weaker coordination with MTMS for all considered electrolytes except for the LCE. Analogous to the previous experimental methods to analyze coordination environments, ^19^F chemical shifts also suggest stronger ion agglomeration and a separate or slowly exchanging coordination environment in MTBE‐based LCE, which cannot be observed in MD simulations. Expanding beyond the interpretation of the chemical shift, ^7^Li and ^19^F pulsed‐field gradient (PFG) NMR enable characterization of the ion mobility via NMR self‐diffusion coefficients (Figure S10a). In the context of ion agglomeration, the ratio of the corresponding Li^+^ (^7^Li) and FSI^−^ (^19^F) NMR self‐diffusion coefficients is of higher interest than their actual magnitude, which correlates with the electrolyte's viscosity^[^ ^59^ ^]^ and is thus not comparable between different electrolytes. Nevertheless, the actual values of the experimentally determined diffusion coefficients can be used to validate the MD simulations (see Figure S10 and Table S5). Once more, predicted and experimental diffusion coefficients exhibit similar trends for the various electrolyte formulations. However, limitations of the non‐polarizable force field employed for MD simulations become evident at higher salt‐to‐solvent ratios, where polarization interactions dominate,^[^ ^60^ ^]^ and thus deviations from experimentally obtained values increase up to one order of magnitude. Regarding the ratio of cationic and anionic diffusion coefficients, the trend for DME‐based electrolytes is closely related to the salt‐to‐solvent ratios. While increased mobility of FSI^−^ is observed in DME‐based LCE and MCE, the respective HCE and LHCE display identical Li^+^ and FSI^−^ diffusion coefficients due to ion agglomeration and correlated diffusion. With MTBE and MTMS as the electrolyte solvent, correlated diffusion of ions is observed at all salt‐to‐solvent ratios.

Summarizing the computational and experimental characterization of electrolyte structures, anionic participation within the lithium solvation sheath is concentration‐dependent with strongly coordinating DME but almost concentration independent for weakly solvating electrolytes containing MTBE and MTMS. Here, steric hindrance introduced by the *tert*‐butyl and trimethylsilyl substituents already limits the coordination strength. While electronic effects caused by the incorporation of silicon further reduce the coordination strength, participation of anions in the lithium coordination sheath is only slightly enhanced. Nevertheless, the low Lewis basicity of both weakly coordinating electrolyte solvents enables higher degrees of ion agglomeration compared to DME‐based electrolytes at all salt‐to‐solvent ratios, even for the HCEs (≥77% versus 72% Li(FSI)_≥2_). Contrary to the local coordination environment, the global structure of lithium clusters in weakly solvating electrolytes depends on the salt‐to‐solvent ratio. While ionic clusters are dispersed in excessively weak solvating electrolytes at low concentrations, their high abundance in high‐concentration electrolyte formulations results in larger cluster sizes. However, a percolating network similar to DME‐based HCE involving 95% of ions cannot be achieved with either weakly solvating electrolyte (Figure 5a). Since further increasing the steric demand of MTMS by prolonging the alkyl groups (yielding ethoxytrimethylsilane) results in an incapability to dissolve the conducting salt, the coordination strength of MTMS can be summarized as very limited and close to non‐coordinating electrolyte solvents.

### Electrochemical Characterization

**Figure 6:**
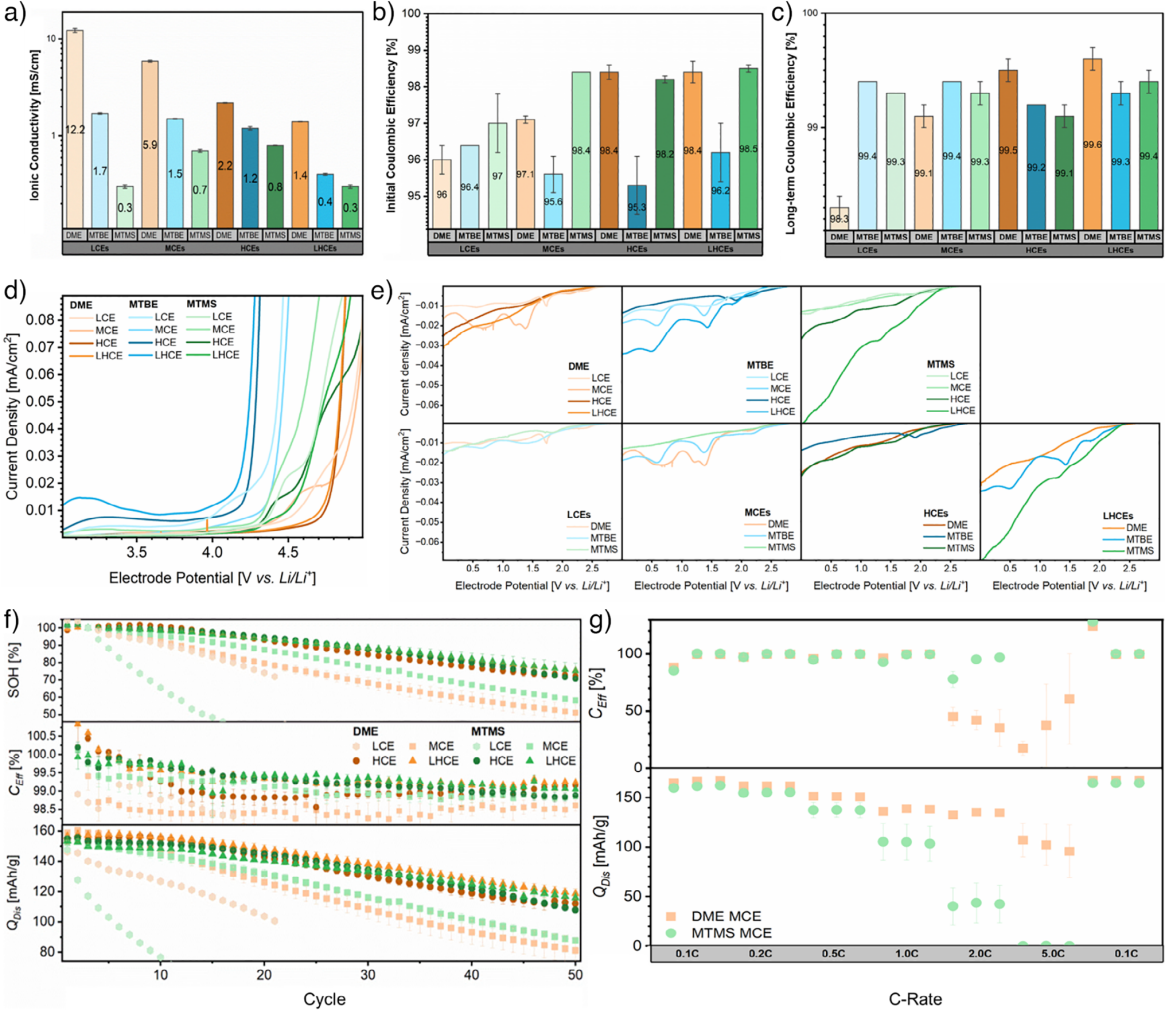
Electrochemical characterization of electrolytes. a) Average ionic conductivity of the considered electrolytes determined from electrochemical impedance spectroscopy measurements. Error bars represent the standard deviation from at least three nominally identical measurements. b) Averaged Coulombic efficiencies for the initial cycle and c) repeated lithium deposition and dissolution in Li‖Cu cells operated with different electrolytes. Error bars represent the standard deviation from at least two nominally identical cells. d) Oxidative stabilities of the respective electrolytes determined in a three‐electrode cell setup against a platinum electrode. e) Reductive stabilities of the respective electrolytes in a three‐electrode cell setup against a copper electrode for identical solvents at varying salt‐to‐solvent ratios (top) and varying solvents at identical salt‐to‐solvent ratios (bottom). f) Electrochemical performance of different DME‐ and MTMS‐based electrolytes in “anode‐free” NMC622‖Cu coin cells. Error bars represent the standard deviation of at least two nominally identical cells. g) C‐rate test for MCEs in NMC622‖Li cells. Error bars represent the standard deviation of at least two nominally identical cells.

Since ion clustering is desirable for anion‐derived interphases of enhanced electrochemical stability but also limits the mobility of ions and thus their vehicular transport,^[^ ^50^, ^54^ ^]^ a trade‐off between reversibility and ionic conductivity is often encountered.^[^ ^15^, ^28^, ^30^, ^51^ ^]^ Furthermore, simultaneous coordination of multiple lithium ions by one anion results in incapability of coupled migration, also increasing the share of structural ionic transport.^[^ ^61^ ^]^ Here, interconnection of lithium coordination sites is of more relevance than ionic mobility,^[^ ^50^, ^53^, ^54^ ^]^ as perceivable from comparison of ionic conductivity for DME‐based HCE and less viscous LHCE (2.2 versus 1.4 mS cm^−1^, Figure 6a). Compared to DME‐based electrolytes, weakly solvating MTBE‐ and MTMS‐based electrolytes display lower ionic conductivities at all salt‐to‐solvent ratios. For the HCEs and LHCEs, this can be reasoned with the size of ionic clusters (Figure 3). Since DME‐based MCEs and LCEs are mostly comprised of solvent‐separated ion pairs (Figures 2 and 4) and therefore can rely on vehicular ion transport, the differences in ionic conductivity compared to MTBE‐ and MTMS‐based electrolytes become more pronounced with decreasing salt‐to‐solvent ratios. Note that this is observed despite the lower electrolyte viscosities of the weakly solvating electrolytes, which is an unavoidable side effect from evaluating mono‐ and bidentate solvents at identical salt‐to‐oxygen moiety ratios (Figure S1). Comparing the ionic conductivity between weakly solvating electrolytes, different trends are observed with increasing salt‐to‐solvent ratios. While the ionic conductivity of MTMS‐based electrolytes scales with the size of ionic clusters and thus is the highest for the HCE, MTBE‐based electrolytes increase their ionic conductivity with lower salt‐to‐solvent ratios. Since contact ion pairs are more prominent in MTBE‐based LCE and MCE, an increased proportion of vehicular transport could explain the inverse trends and diminished dependence on ion cluster size for MTBE‐based electrolytes.

With weakly solvating MTBE‐ and MTMS‐based electrolytes displaying lower ionic conductivities, questions arise regarding their applicability in cells operated at typically relevant charge and discharge rates. Here, it should be noted that enhancing the ionic conductivity by combining various conducting salts and diluents to form high‐entropy electrolytes^[^ ^43^, ^62^ ^]^ afforded no improvement of ionic conductivity for the MTMS‐based LHCE (Supplementary Note 1). To assess the impact of weakened solvation strength on both the ionic transport and reversibility of lithium metal inventory, LiǁCu cells were operated with different electrolytes utilizing a standardized protocol.^[^ ^63^, ^64^ ^]^ While all the electrolytes enable sufficient ionic transport to be operated at a current density of 0.5 mA cm^−2^, the overpotentials for lithium metal deposition and dissolution (Figure S11) align with trends in ionic conductivity. Regarding the reversibility of lithium metal inventory, a correlation to the extent of anion participation in the lithium solvation sheath is readily observable. While weakly solvating MTBE‐ and MTMS‐based electrolytes display similar average Coulombic efficiencies at all salt‐to‐solvent ratios (Figure 6c), strongly solvating DME‐based MCE and especially LCE with lower anionic coordination numbers exhibit notably deteriorated reversibility. However, the local coordination environment seems to not be the only factor having an impact on the lithium reversibility. Though increased participation of anions was observed in MTBE‐ and MTMS‐based HCEs and LHCEs, DME‐based HCEs and LHCEs enable the highest reversibility of lithium inventory. Apparently, the enhanced ionic conductivity and thus lower overpotentials for lithium metal deposition and dissolution (Figure S11) have a positive impact on the reversibility of lithium inventory. Nevertheless, for eco‐friendly electrolyte formulations that do not rely on the presence of per‐fluorinated solvents or diluents and have moderate conducting salt concentrations, the weakly coordinating electrolytes containing MTBE and MTMS boost the reversibility of lithium inventory relative to the corresponding benchmark electrolytes. Comparing the long‐term reversibility of lithium inventory with either MTBE‐ or MTMS‐based electrolytes reveals little impact of silicon substitution, with Coulombic efficiencies (*C_Eff_*) being similar (within error margins) for both weakly solvating electrolytes at all salt‐to‐solvent ratios. In the formation cycle (Figure 6b), however, differences between MTMS‐ and MTBE‐based electrolytes can be observed. Here, MTBE‐based electrolytes display more irreversible capacity loss at every salt‐to‐solvent ratio, indicative of an altered initial interphase formation. Furthermore, MTBE‐ and MTMS‐based electrolytes also exhibit differences in the evolution of lithium deposition and dissolution overpotentials (Figure S11). While these remain constant or even increase in the case of MTBE‐based electrolytes, a stabilization of interphases indicated by a decrease in overpotential is observed with every considered MTMS‐based electrolyte formulation. Thus, lithium metal deposition and dissolution overpotentials in MTMS‐based electrolytes are lower than in cells cycled with MTBE‐based electrolytes after 10 cycles, despite lower ionic conductivities. To determine whether the weakly solvating electrolytes may also be employed in application‐oriented cells, their electrochemical stability window is characterized by linear sweep voltammetry in custom‐made three‐electrode cells. While reduction processes at the copper electrode provide further insights on the initial interphase formation at negative electrodes (Figure 6e), sufficient oxidative stability against the platinum electrode is essential for positive electrode compatibility (Figure 6d). Especially for batteries operated with nickel–manganese–cobalt (NMC) positive electrodes, which are typically charged to cut‐off voltages ≥4 V, the lone electron pair of the oxygen moiety in ether‐based electrolytes can be prone to oxidation.^[^ ^21^ ^]^ While strongly coordinating solvents achieve enhanced oxidative stability at high conducting salt concentrations due to distribution of electron density toward Li^+^ and the absence of uncoordinated solvent molecules,^[^ ^21^, ^65^ ^]^ achieving sufficient oxidative stability for weakly coordinating solvents can be challenging.^[^ ^22^, ^34^ ^]^ Here, the dissemination of electron density toward lithium ions through coordination is less pronounced. Thus, the electrolytes' oxidative stability is limited by the intrinsic oxidative stability of the solvents, even at high salt‐to‐solvent ratios.

This is reflected by the oxidative stability of all MTBE‐based electrolyte formulations, with decomposition reactions starting at 4.0 V versus Li/Li^+^ and a steep rise in oxidative current in the region of 4.2–4.3 V versus Li/Li^+^ (Figure 6d). In case of strongly coordinating DME as the electrolyte solvent, oxidative currents for HCE and LHCE are detected starting from 4.6 V versus Li/Li^+^. At lower concentrations (MCE and LCE), increased availability of non‐coordinating DME molecules (Figure S9) not being stabilized by electron donation toward Li^+^ decreases the onset of oxidative decomposition to 4.3 V versus Li/Li^+^.^[^ ^21^ ^]^ With MTMS instead of MTBE as the electrolyte solvent, the oxidative stability is enhanced at every salt‐to‐solvent ratio. Despite also being limited in coordination strength, incorporation of silicon shifts the actual onset of oxidative decomposition from 4.0 V versus Li/Li^+^ for MTBE‐based electrolytes to 4.3 V versus Li/Li^+^ for MTMS‐based electrolytes. This enhanced oxidative stability aligns with the atomic charges and orbital populations determined by DFT calculations (Figure 1b,c), where negative hyperconjugation effectively decreases the electron population in the oxygen lone‐pair orbital. Fittingly, the oxidation potentials based on the highest occupied molecular orbitals (HOMOs, 9.94 eV versus 9.73 eV) as well as changes in Gibbs free energy upon oxidation (7.79 eV versus 7.39 eV) are also increased when comparing MTMS and MTBE molecules. Hence, altered distribution of electron densities when incorporating silicon constitutes a highly effective strategy to increase the oxidative stability of weakly solvating electrolytes. In view of reductive stability, the anion‐rich coordination environments of all MTMS‐based electrolytes exhibit similar decomposition potentials (Figure 6e). Accordingly, the reductive peaks of DME‐based HCE and LHCE, previously characterized to also exhibit high anion participation in lithium coordination spheres, align well with MTMS‐based electrolytes. With less ion agglomeration in DME‐based MCE and particularly LCE, the reductive electrolyte decomposition begins at higher potentials. This correlates well with lowest occupied molecular orbital (LUMO) calculations reported for solvent‐ and anion‐dominant lithium coordination clusters, where the molecular orbitals for the former are of lower energy (more stabilized) and thus can be populated (reduction of the coordination cluster) at less negative (higher) potentials.^[^ ^22^ ^]^ With MTBE instead of MTMS as the electrolyte solvent, the reductive voltammograms differ notably, despite similar lithium coordination clusters. These differences in the initial interphase formation of weakly solvating electrolytes are also observed in LiǁCu cells (Figure 6b), exhibiting a diminished reversibility for all considered MTBE‐based electrolytes. Though this indicates additional irreversible reduction processes in MTBE‐based electrolytes, reductive potentials for uncoordinated MTMS and MTBE based on Gibbs free energy differences (−0.02 eV versus −0.07 eV) as well as LUMO energies (−1.03 eV versus −1.08 eV) suggest MTBE to be slightly more stable reductively. In contrast to similar HOMO distributions (Figure 1b), spatial arrangements of the LUMO in MTMS and MTBE differ. For MTMS, the LUMO is more diffuse and also located near the oxygen moiety (Figure S12), which could potentially lead to altered decomposition pathways and products. Nevertheless, transferability of these computational insights to the actual reductive electrolyte decomposition in cells is limited since coordination environments and their respective geometry,^[^ ^22^ ^]^ as well as previous decomposition reactions, should also be considered.^[^ ^66^ ^]^ To characterize the reversibility of the selected electrolytes in the context of full‐cell operation, NMC‐based “anode‐free” or “zero‐excess” lithium metal batteries represent favorable cell configurations. Here, the compatibility with commonly utilized high‐voltage (>4 V) NMC positive electrodes can be simultaneously evaluated with the reversibility of lithium inventory (Figure 6f), which is often overshadowed by the excessive lithium metal reservoir present in conventional lithium metal batteries.^[^ ^1^, ^3^ ^]^ Due to the low oxidative stability of MTBE‐based electrolytes, oxidative electrolyte decomposition impedes reversible cell operation, as shown by a voltage plateau at 4.15 V (Figure S13). In contrast, MTMS‐based electrolytes can be operated in “anode‐free” lithium metal batteries due to negative hyperconjugation increasing the intrinsic oxidative stability of the electrolyte solvent. Nevertheless, cells operated with MTMS‐based LCE display rather fast capacity decay. Since this electrolyte exhibits facile long‐term reversibility for deposition and dissolution of lithium metal in LiǁCu cells (99.3% average Coulombic efficiency, Figure 6c), we attribute the low reversibility in NMC‐based cells to parasitic reactions at the positive electrode, despite comparable oxidative stability observed for all MTMS‐based electrolytes in linear sweep voltammetry (LSV) measurements. In fact, such differences in oxidative stability against model (Pt) and real (NMC622) electrodes are known and can be reasoned with varying active area and chemical reactivity of such electrodes.^[^ ^67^ ^]^ Since cells with DME‐based LCE display a short circuit after 22 cycles, neither LCE is suitable for full cell operation. Moderately concentrated electrolytes, however, can be reversibly operated in “anode‐free” lithium metal batteries with both DME and MTMS. Though the reduced ionic conductivity and thus increased overpotential of MTMS‐based MCE lowers the initial discharge capacity, better long‐term capacity retention is achieved with the enhanced ion agglomeration of the weakly solvating electrolyte. This is despite lower conducting salt concentration (1.5 mol kg^−1^ instead of 2.5 mol kg^−1^ when comparing MCEs, Figure S1) due to the approach of maintaining identical salt‐to‐oxygen moiety ratio between mono‐ and bidentate solvents. Comparing the electrolyte solvents at similar LiFSI concentrations (1.6 mol kg^−1^ in DME LCE and 1.5 mol kg^−1^ in MTMS MCE, Figure S1) reveals an even more pronounced enhancement of reversibility with MTMS instead of DME as the electrolyte solvent. Evoking the concept of HCEs or LHCEs, the reversibility of lithium inventory is improved compared to moderate concentration electrolytes, as already observed in LiǁCu cells. Here, the choice of electrolyte solvent has negligible implication on the electrochemical performance, while the effective conducting salt concentration is lower with MTMS. However, focusing on eco‐friendly and cost‐competitive electrolytes, MTMS as a weakly coordinating solvent not only enables lower conducting salt concentrations but also boosts the reversibility of lithium inventory. As indicated by the limited ionic conductivity (Figure 6a), these advantages come at the expense of a decreased rate capability. To also compare ionic transport in weakly solvating and reference electrolyte in an application‐oriented cell configuration, the achievable discharge capacity for NMC positive electrodes operated with DME‐ and MTMS‐based MCEs is determined with consecutively higher operating rates, commonly referred to as a C‐rate test (Figure 6g). Here, lithium metal negative electrodes are employed to avoid that reversibility of lithium inventory becomes a limiting factor for the achievable discharge capacities. While the decrease in specific discharge capacities with MTMS instead of DME‐based MCE is tolerable (≤ 10%) at rates of up to 0.5C, they are more pronounced with increasing currents, resulting in 24% and 68% less specific discharge capacity compared to the reference system at 1.0C and 2.0C, respectively. Though cells with DME‐based MCE also deliver diminished capacities with increasing operation rates, they can still be operated at 5.0C, whereas insufficient ionic transport with MTMS‐based MCE prevents cells from being cycled at this rate. However, despite sufficient ionic transport, the DME‐based MCE is not suitable for cell operation beyond 1.0C. At 2.0C and 5.0C, reversibility becomes a limiting factor, as indicated by a notable decrease in Coulombic efficiencies. Similar to MTBE‐based electrolytes, the increase in charge capacity can be ascribed to oxidative electrolyte decomposition based on the respective voltage profiles (Figure S14). Since no excessive decomposition is observed at operating rates below 2.0C, the oxidative stability of nonsubstituted ethers seems to depend on the operating rate. In summary, the C‐rate test vividly demonstrates the limitations of both moderately concentrated electrolytes. While MTMS‐based MCEs do not offer sufficient ionic conductivity to be operated at high C‐rates, DME‐based MCEs are limited by their oxidative stability. Here, continued operation of NMCǁLi cells after a C‐rate test (Figure S15) still indicates enhanced reversibility in the presence of weakly solvating electrolytes. However, subsequent analysis of lithium metal deposit morphology via scanning electron microscopy (SEM, Figure S16) reveals compact as well as mossy lithium deposits to be present in the case of both MCEs.

## Conclusion

**Figure 7:**
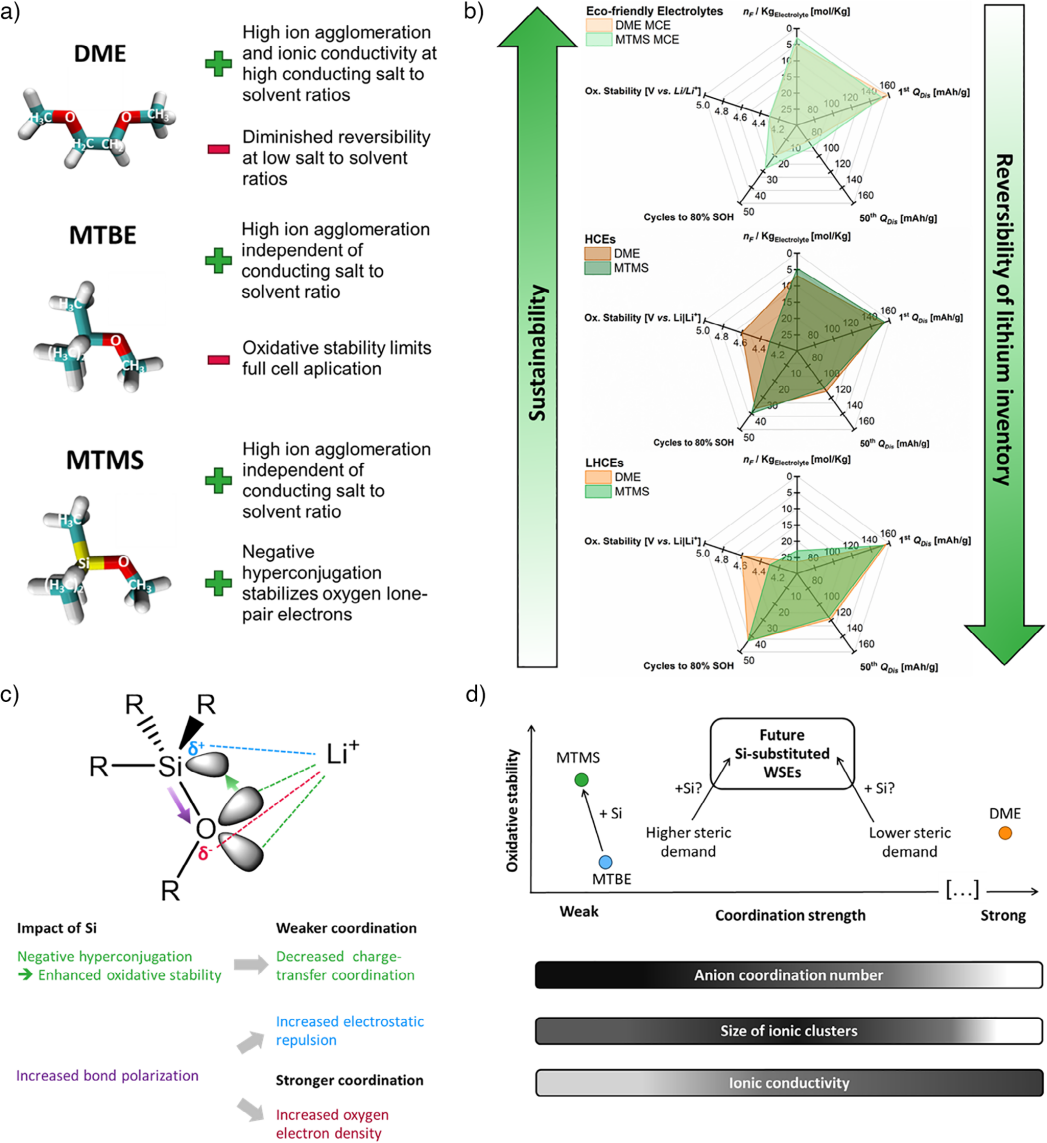
a) Advantages and drawbacks of the compared electrolyte solvents. b) Spider diagrams comparing key performance indicators of NMC622ǁCu cells operated with different electrolytes. c) Interplay of steric and electronic effects in silicon‐substituted ethers. d) Classification of electrolyte solvents characterized herein and proposed electrolyte design strategies for future efforts.

To broaden the scope for eco‐friendly electrolytes in high‐energy‐density batteries, silicon substitution was herein evaluated as a strategy to design weakly coordinating electrolyte solvents. Here, the increased bond polarization due to the lower electronegativity of silicon compared to carbon had an ambiguous effect. While increasing electron density at oxygen, it also enhanced static repulsion of lithium ions from positively polarized silicon. Negative hyperconjugation as an additional effect from incorporation of silicon successfully dispelled electron density of the oxygen lone pair, as confirmed by density functional theory calculations. While the interplay of these effects decreased the coordination strength when incorporating silicon in methyl *tert*‐butyl ether, molecular dynamic simulations as well as Raman and nuclear magnetic resonance spectroscopy indicated that anionic coordination environments are already generated by the steric demand of the *tert*‐butyl substituent. Since incorporation of silicon only resulted in a minor additional increase of anionic lithium coordination, both weakly solvating electrolytes displayed similar long‐term reversibility of lithium inventory in LiǁCu cells. Initial interphase formation, however, was found to be more reversible in the case of silicon‐substituted electrolyte solvents. Moreover, the introduced electrolytes exhibited enhanced oxidative stability, which was not only observed using linear sweep voltammetry against a platinum electrode but also in application‐oriented cells operating with higher voltage nickel–manganese–cobalt (NMC) positive electrodes. While methyl *tert*‐butyl ether electrolytes could not be reversibly operated due to oxidative decomposition of the electrolyte solvent, negative hyperconjugation with the antibonding Si─C orbital (σ*Si─C) dispelled electron density from the solvent's oxygen lone‐pair orbital, thereby stabilizing the highest occupied molecular orbital and enabling cell operation beyond 4 V. Contrary to the strongly coordinating reference solvent 1,2‐dimethoxyethane, both weakly solvating electrolytes achieved participation of anions in the lithium coordination spheres independent of the salt‐to‐solvent ratio (Figure 7a). However, the global electrolyte structure, referring to the interconnection of these ionic clusters, correlated with the salt‐to‐solvent ratio for weakly solvating electrolytes. Since a percolating network of coordination sites facilitates ion transport in agglomerated electrolyte structures and was maximized at high salt‐to‐solvent ratios with either electrolyte solvent, the electrochemical performance of (localized) high‐concentration electrolytes remained unmatched (Figure 7b). Under these conditions, weakening the solvent coordination strength offered no merits, since ion agglomeration is forced by low availability of solvent molecules anyway. Instead, a lower cluster size with weakly coordinating solvents was found to be detrimental for the ionic conductivity and thus overpotentials for lithium deposition and dissolution, thereby limiting reversibility and rate capability.

Nevertheless, with high conducting salt concentrations driving electrolyte costs and per‐fluorinated alkyl substances potentially facing bans, (localized) high‐concentration electrolytes have little room for industrial application. Under eco‐friendly and cost‐competitive conditions, the herein characterized weakly coordinating electrolyte solvent methoxytrimethylsilane clearly outperformed the benchmark electrolyte, despite a lower conducting salt concentration. Here, the tailored coordination strength of the weakly solvating electrolytes achieved participation of anions in the lithium solvation sheath at moderate or low concentrations, boosting the reversibility of lithium inventory. However, solely the increased oxidative stability due to negative hyperconjugation (Figure 7c) allowed to transfer the enhanced reversibility to application‐oriented cell chemistries. Compared to previous approaches stabilizing weakly coordinating electrolyte solvents via inductive effects from fluorine substituents, incorporation of silicon represents an affordable and eco‐friendly approach. Nevertheless, C‐rate tests revealed impeded ionic transport as a limiting factor for the rate capability of the introduced weakly coordinating electrolyte. Also, methoxy‐trimethylsilane has high vapor pressure and flammability, which should be addressed in further work to render siloxanes commercially viable electrolyte solvents. Thus, transfer of the herein evaluated design principle to other ether‐based structures is suggested as an important next step. Here, the objective is to tailor the coordination strength in a way that anions participate in the lithium solvation sheath while not overly sacrificing the achievable ionic conductivity. As observed in the analysis of cluster sizes, larger networks of coordination sites and thus enhanced ionic conductivities are obtained with methyl *tert*‐butyl ether‐based electrolytes, the weakly solvating electrolyte solvents with higher Lewis basicity. While incorporation of silicon reduced the coordination strength in this case, computational data of silicon‐substituted dimethoxyethane analogues also indicated their potential to enhance the coordination strength. Thus, next to incorporating silicon to enhance the oxidative stability of ether‐based electrolytes, we suggest further employing the interplay of steric and electronic effects for tailoring solvent molecular structures (Figure 7d). Starting from structural motives that have little steric limitation, incorporation of silicon can be utilized to further decrease coordination strength, as demonstrated for methyl *tert*‐butyl ether in this work. For solvent molecules that are already weakly coordinating due to high steric demands of substituents, higher bond polarization in the presence of silicon should be used to slightly enhance coordination strength, achieving a favorable compromise of both ion agglomeration and ionic conductivity.

## Supporting Information

The authors have cited additional references within the Supporting Information.^[^ ^43^, ^44^, ^45^, ^62^, ^63^, ^64^, ^68^, ^69^, ^70^, ^71^, ^72^, ^73^, ^74^, ^75^, ^76^, ^77^, ^78^, ^79^, ^80^, ^81^, ^82^, ^83^, ^84^, ^85^, ^86^, ^87^, ^88^, ^89^, ^90^ ^]^

## Conflict of Interests

The authors declare no conflict of interest.

## Supporting information



Supporting Information

## Data Availability

The data that support the findings of this study are available from the corresponding author upon reasonable request.
