# Supplementary material for: Design of Fluorine‐Free Weakly Coordinating Electrolyte Solvents with Enhanced Oxidative Stability
Source: Angew Chem Int Ed Engl. 2025 Jul 20;64(35):e202506826. doi: 10.1002/anie.202506826 (PMC12377438; doi:10.1002/anie.202506826)
Supplement: Supplementary file 1 — Supporting Information [file ANIE-64-e202506826-s001.pdf]

Supporting Information: Design of Fluorine-Free Weakly Coordinating Electrolyte Solvents with Enhanced Oxidative Stability

Lennart Wichmann[a], Adil Aboobacker[a‡] Steffen Heuvel[b,c ‡], Felix Pfeiffer[a], Robert-Tobias Hinz[a], Frank Glorius[c], Isidora Cekic-Laskovic[a], Diddo Diddens[a], Martin Winter[a,d], Gunther Brunklaus[a*],

a Helmholtz –Institute Münster, IMD-4, Forschungszentrum Jülich GmbH, Corrensstr. 48, 48149 Münster, Germany

b International Graduate School for Battery Chemistry, Characterization, Analysis, Recycling and Application (BACCARA), University of Münster, Corrensstr. 40, Münster 48149, Germany

c Institute of Organic Chemistry, University of Münster, Corrensstraße 36, 48149 Münster, Germany

d MEET Battery Research Centre, Institute of Physical Chemistry, University of Münster, Corrensstr. 46, 48149 Münster, Germany

*Corresponding author: g.brunklaus@fz-juelich.de (G.B.), ‡ these authors contributed equally.

Experimental

DFT Calculations

Density functional theory (DFT) calculations were performed using the ORCA quantum chemistry software package. [1,2] Geometry optimizations were done using ωB97M-V [1] functional with the def2-TZVPD basis set. [3] Tight self-consistent field (SCF) convergence criteria were chosen. Solvation effects were modeled using the conductor-like polarizable continuum model (CPCM) [4] with tetrahydrofuran (THF) as the solvent, exhibiting a similar dielectric constant as the solvents employed in this study. Atomic charges were derived employing the CHELPG (Charges from electrostatic potentials using a grid-based method) scheme. [5] The Pipek-Mezey (PM) method [6] was utilized for orbital localization, facilitating the identification of lone pair electron densities. Natural Bond Orbital (NBO) analysis was performed using Gaussian 16 [7], employing the ωB97X-D functional [8] and the aug-cc-pVTZ [9,10] basis set. NBO version 6.0 [11] was used, as implemented in Gaussian, to evaluate non-Lewis (*i.e.*, delocalized) orbital occupancies for quantifying negative hyperconjugative interactions.

Molecular Dynamics Simulations

All molecular dynamics (MD) simulations were performed using the GROMACS simulation package. [12] Ion charges were scaled by a factor of 0.8 to account for polarization effects in high-concentration systems. [13] The OPLS-AA non-polarizable force field [14] was employed, parameters for MTMS, MTBE and TTE were generated using LigParGen [15] and the rest were taken from reported literature. [14,16] The charges for MTMS were replaced with ChelpG charges obtained from DFT and further scaled to match the dielectric constant of MTMS and MTBE at room temperature (**Table S1**).

Table S1: Scaled charges for MTMS.

Element	Charge	Element	Charge	Element	Charge
C	-0.153996	H	0.082915	H	0.221522
O	-0.548267	H	0.082836	H	0.221120
Si	1.304839	H	0.130615	H	0.221059
C	-0.749048	H	0.153591	H	0.153631
C	-0.995638	H	0.146410	H	0.165464
C	-0.749056	H	0.165569	H	0.146434

The MTMS-based LHCE exhibited an abnormally high degree of ion clustering (**Figure S3**), which could potentially be mitigated by further scaling down the ion charges. However, in this study, we focus on binary-solvent systems (LCEs, MCEs, and HCEs).

Initial molecular configurations were prepared using Packmol [17] randomly distributing 100 Li^+ ions, 100 FSI^- ions and corresponding number of solvent/eluent molecules in the defined ratios (**Figure S1** and **Table S2**) in a box. For each system, an energy minimization at T = 0 K was carried out, then it was heated in a number-pressure-temperature (NPT) ensemble to 373 K under 2 bar pressure using the Berendsen thermostat to ensure homogeneous mixing. The systems were then equilibrated in an NPT ensemble for 20 ns at 300 K and production runs were performed in a number-volume-temperature (NVT) ensemble for 250 ns to evaluate the diffusion coefficients. Further analyses were conducted using custom scripts and the MDAnalysis library [18,19] to find average coordination numbers and quantify the aggregation of ion pair clusters. The average ion pair coordination numbers were obtained by analyzing the last 100 frames of the production run and taking the average number of Li-N pairs within a cut-off of 5 Å (**Figure S3**).

To investigate clustering, we first examined the Li–Li radial distribution function (RDF) in each system and identified the distance of 7 Å (**Figure S6**) for the first peak as the cut-off criterion for cluster formation. This cut-off was used to determine Li–Li connectivity in representative snapshots from the production run. Any two Li atoms separated by less than or equal to the cut-off were considered to

belong to the same cluster. Using this single-linkage approach, we subsequently calculated both the population and size distribution of Li clusters.

Table S2: Number of conducting salt and electrolyte solvent molecules in MD simulations of all electrolyte formulations.

Electrolyte	LiFSI Molecules	DME Molecules	MTBE Molecules	MTMS Molecules
DME LCE	100	480	-	-
DME MCE	100	240	-	-
DME HCE	100	120	-	-
MTBE LCE	100	-	960	-
MTBE MCE	100	-	480	-
MTBE HCE	100	-	240	-
MTMS LCE	100	-	-	960
MTMS MCE	100	-	-	480
MTMS HCE	100	-	-	240

Materials and Electrodes

NMC-based composite electrode slurry was cast onto aluminum foil (15 µm, Nippon) using a coating machine (HSCM-20802i, Hohsen Corp.), at a wet film thickness of 59 µm. In a typical approach, the electrode paste resulted from mixing 95 wt.% NMC622 (BASF), 3 wt.% PVdF1100 (Kureha) and 2 wt.% SuperC65 (TIMCAL/Imerys) in *N*-methylpyrrolidine (Sigma-Aldrich) in a planetary mixer (Eirich), using a solid to liquid ratio of 77:23. After drying the cast electrode at 100 °C and subsequently at 120 °C, both for 12 h, all the resulting electrodes (with active material mass loadings of 13.5 mg/cm^2 and areal capacity of 2.3 mAh/cm^2) were calendared (GKL 400, Saueressig Group) to a thickness of 57 µm and a resulting total porosity ε of 30%, according to literature [20,21].

A copper sheet (Schlenk Metallfolien GmbH, 10 µm) was immersed in glacial acetic acid (99.9%, Sigma-Aldrich) for 10 min. Upon removing the copper sheet from the acetic acid bath, remaining acetic acid was evaporated homogeneously by exposure to an Argon gas stream in dry room atmosphere (with dew point of -55 to -60 °C).

The considered electrolytes were formulated by mixing the conducting salt LiFSI (Elyte Innovations, battery grade) with either 1,2-dimethoxyethane (DME, Sigma Aldrich, anhydrous, 99.5%), methyl *tert*-butyl ether (MTBE, Sigma Aldrich, 99%) or methoxytrimethylsilane (MTMS, Sigma Aldrich, 99%) in the molar ratio stated in **Figure S1**. To obtain the LHCEs, 1,1,2,2-tetrafluoroethyl-2,2,3,3-

tetrafluoropropylether (TTE, SynQuest Laboratories, 97%) was added subsequently according to the molar ratio given in **Figure S1**. Since these solvents were not commercially available in anhydrous quality, TTE, MTBE and MTMS were dried 24 h over vacuum-dried molecular sieves (0.3 – 0.4 nm, Sigma Aldrich) prior to use. To avoid electrolyte contamination by solid residues of molecular sieves, the solvents were only added using hydrophobic syringe filters (Sigma Aldrich).

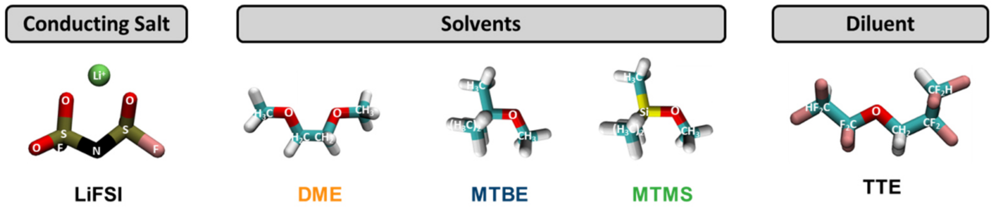

	Low Concentration			Moderate Concentration			High Concentration			Localized High-Concentration		
LiFSI	DME	MTBE	MTMS	DME	MTBE	MTMS	DME	MTBE	MTMS	DME:TTE	MTBE:TTE	MTMS:TTE
1 :	4.8	9.6	9.6	2.4	4.8	4.8	1.2	2.4	2.4	1.2:3	2.4:3	2.4:3
c_{LiFSI} [mol/kg]	1.6	1.0	0.8	2.5	1.6	1.5	3.4	2.5	2.3	1.0	0.9	0.9
c_F [mol/kg]	3.2	1.9	1.7	5.0	3.3	2.9	6.8	5.0	4.6	26.3	23.7	22.9

Figure S1: Overview of electrolyte constituents and formulations employed in this work. While c_{LiFSI} refers to the molar concentration of LIFSI in one kg of the respective electrolyte formulation, c_F is defined as the molar concentration of fluorine atoms in one kg of the respective electrolyte. Here, every LiFSI molecule accounts for two fluorine atoms and every TTE molecule accounts for eight fluorine atoms.

Assembly and operation of Li||Cu cells

For the assembly of Li||Cu cells, 12 mm Ø lithium metal electrodes (300 µm thick Li^0, Honjo Lithium) employed as positive electrode were separated from 15 mm Ø copper electrodes, (see materials) using a 16 mm polyethylene separator (Celgard 2500) wetted with 30 µL of the respective electrolyte in CR2032 coin cells (TOB NewEnergy). The cell stack was then sandwiched between 0.5 mm thick stainless-steel spacer (TOB NewEnergy), a 1.2 mm thick stainless-steel wave spring (TOB NewEnergy) and a 1.0 mm thick stainless-steel spacer (TOB NewEnergy). All the cells were assembled in an argon-filled glovebox (MBraun, < 0.5 ppm O_2, < 0.5 ppm H_2O) and sealed using an automated electric crimper (Hohsen Corporation).

For the electrochemical evaluation procedure, a standardized protocol [22,23] with a constant current density of 0.5 mA/cm^2 was invoked. After an initial formation cycle of depositing lithium at the copper electrode with a capacity of 5 mAh/cm^2 and subsequent lithium dissolution until a -1.0 V cut-off voltage was reached, a lithium reservoir of 5 mAh/cm^2 was deposited onto copper. In Li||Li@Cu configuration, ten cycles of repeated lithium metal dissolution and deposition of 1 mAh/cm^2 was carried out, followed by a final lithium dissolution until a -1.0 V cut-off voltage occurred. The long-term Coulombic efficiency was derived by relating the overall lithium metal dissolution capacity to the deposition capacity after the initial formation cycle. All the procedures were carried out at a Maccor Series 400 battery cycler (Maccor Incorporation) at the temperature of 20 °C.

Assembly and operation of NMC622||Cu cells

Likewise, for the assembly of NMC622||Cu cells, 14 mm Ø NMC622 electrodes as the positive electrode were separated from 15 mm Ø copper electrodes (see materials) using a 16 mm polyethylene separator (Celgard 2500) wetted with 30 µL of the respective electrolyte in CR2032 coin cells (TOB NewEnergy). To avoid stainless-steel dissolution in the presence of LiFSI at cell voltages ≥4 V, an aluminum coated positive cap (MTI) was used. The cell stack was sandwiched between a 0.5 mm thick stainless-steel spacer (TOB NewEnergy) and a 1.2 mm thick stainless-steel wave spring (TOB NewEnergy) at the negative electrode and a 1.0 mm thick aluminum coated spacer (MTI) and a 19 mm Ø aluminum foil at the positive electrode. All the cells were assembled in an argon-filled glovebox (Mbraun, < 0.5 ppm O_2, < 0.5 ppm H_2O) and sealed using an automated electric crimper (Hohsen Corporation). After resting at open circuit voltage for 12 h, all the NMC622||Cu cells were charged at a current density of 0.2 mA/cm^2 for two cycles and at 0.4 mA/cm^2 thereafter. The discharging step was carried out at 0.6 mA/cm^2 for all cycles. The lower and upper cut-off voltages were set to 3.0 V and 4.3 V, respectively. All the electrochemical procedures were carried out on a Maccor Series 400 battery cycler (Maccor Incorporation) at the temperature of 20 °C.

Assembly and operation of NMC622||Li cells

NMC622||Li cells were assembled in a glovebox (Mbraun, < 0.5 ppm O_2, < 0.5 ppm H_2O) analogous to NMC622||Cu cells but with a lithium metal electrode comprised of 20 µm thick lithium metal on 10 µm thick copper (Honjo Lithium) instead of a copper negative electrode. Cells were operated in a voltage range from 3.0 V to 4.3 V, with the charge and discharge C-rate consecutively increasing after 3 cycles. The C-rates were based on a theoretical specific capacity of 160 mAh/g and selected to be 0.1C, 0.2C, 0.5C, 1.0C, 2.0C, 5.0C followed by a recovery at 0.1C. After this C-rate test, cells were operated with a constant current procedure using 0.2C as the charge and 0.3C as the discharge current in the identical voltage window of 3.0 – 4.3 V.

Linear Sweep Voltammetry

Linear sweep voltammetry (LSV) experiments were carried out in custom-made three-electrode T-cells (Swagelok©). For the reductive stability, a 1 mm Ø Cu electrode surrounded by PEEK was used as a working electrode, while for the oxidative stability a 1 mm Ø Pt electrode surrounded by PEEK was utilized as working electrode. For a disassembled view of LSV cells, see **Figure S1**.

Prior to cell assembly, the Pt- and Cu-electrodes were thoroughly rinsed with ethanol and polished with both sandpaper and a 1 mm diamond suspension. All other T-cell components were cleaned with ethanol, water and again ethanol exploiting a sonication bath. After cleaning, all the cell components were carefully dried in a 60 °C temperature chamber (Binder). Subsequently, all cells were assembled

in a glovebox (MBraun, O_2 < 0.5 ppm, H_2O < 0.5 ppm). Prior to cell assembly, the body of three-electrode cells was covered with insulating polyethylene terephthalate foil (PET, 100 µm thickness, Mylar). Lithium metal was used as counter (12 mm Ø, 20 µm thick Li^0 on copper, Honjo Lithium) and reference (8 mm Ø, 600 µm thick Li^0, Honjo Lithium) electrodes, each separated by three-layers of polyolefin separator (525 µm stacking height, Freudenberg FS2190) with 13 and 10 mm Ø respectively. 120 and 80 µL of electrolyte were used to wet the former and later. After assembly, the LSV cells were connected to a VMP-3e multichannel potentiostat (BioLogic) and rested at open circuit potential for 6 h. A scan rate of 0.1 mV/s was applied to the working electrode until the cut-off potential of 1 mV *vs.* Li|Li^+ (Cu electrode, reductive) or 5.0 V *vs.* Li|Li^+ (Pt electrode, oxidative) was reached.

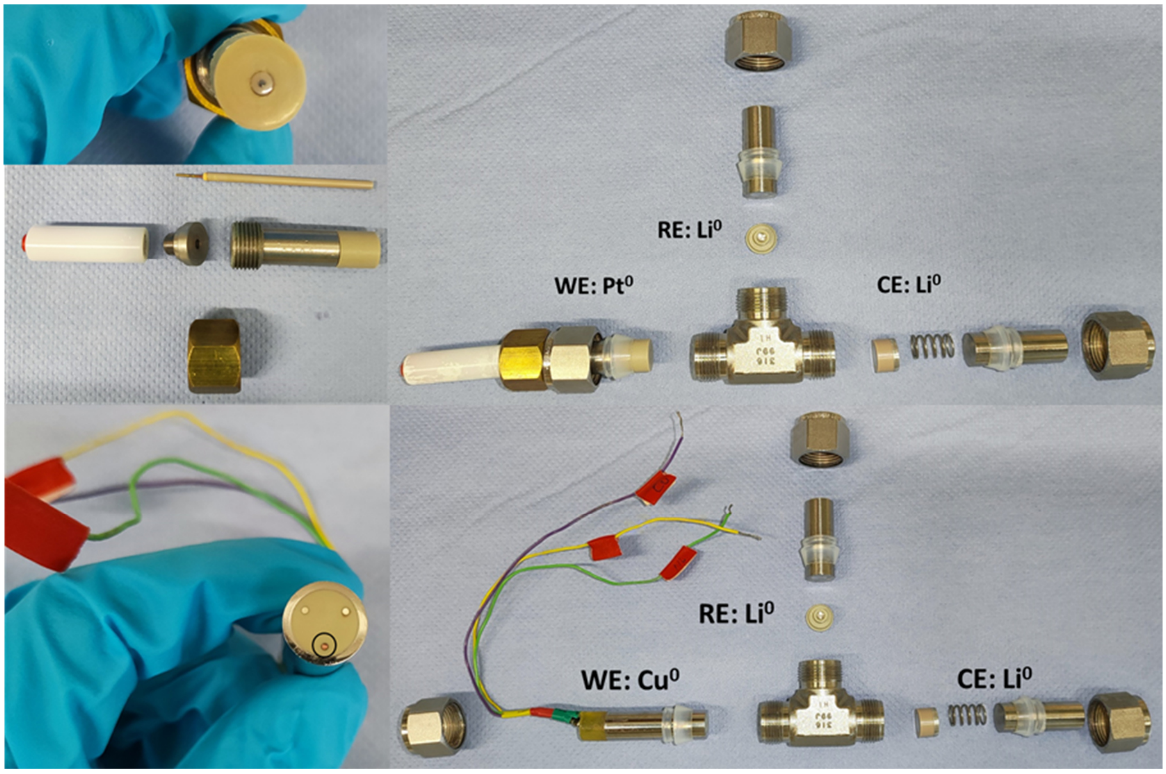

Figure S2: Disassembled view of custom-made three-electrode T-cells for determination of oxidative (top) and reductive (bottom) decomposition of considered electrolytes.

Raman spectroscopy

Raman measurements were conducted using a confocal Raman microscope (Horiba Scientific, LabRAM HR evolution, air-cooled CCD detector with an 1800 g/mm grating). The samples were excited by a red laser (633 nm) with a power output of 10.50 mW at the objective, adjusted to a power to 5.25 mW using a 50% filter. The laser was focused by a 50X long-working distance objective (Carl Zeiss Microscopy, 9.2 mm, numerical aperture 0.5). Raman spectra were recorded by ten integrations of 35 s. The Raman spectrometer, data acquisition, and analysis were handled using LabSpec 6.7.1.10 (Horiba Scientific). Prior to the measurements, the system was calibrated with the peak of crystalline silicon at 520.7 cm^{-1}.

Ionic Conductivity

To determine average ionic conductivity and its standard deviations for all electrolytes, a previously reported high-throughput experimentation approach was utilized [24–26]. In this case, 375 µL of electrolyte was filled into disposable 1.5 mL safe-lock tubes (Eppendorf) in a glovebox atmosphere (MBraun, < 0.5 ppm O_2, < 0.5 ppm H_2O). For each electrolyte formulation, six samples were prepared and placed into the custom sample rack. After sealing the safe-lock tubes air-tight with the custom blocking electrodes and sample rack, electrolyte samples were transferred to a temperature chamber (Memmert TTC256). Here, the temperature was controlled in the range of -30 °C to 60 °C in steps of 10 °C. After equilibrating at the set temperature for 2 h, impedance measurements were carried out with a 40 mV amplitude in the frequency range from 20 kHz to 50 Hz, using a Metrohm Autolab/M204 potentiostat. The electrolyte resistance was obtained by fitting impedance spectra with an R_s(CPE-R_p) equivalent circuit model (**Figure S3**). The quotient of the respective cell constant previously determined with 0.01 M KCl in H_2O at 20 °C (VWR, 1.276 mS/cm) and the electrolyte resistance yielded the temperature dependent ionic conductivity, which was subsequently averaged considering only viable impedance data.

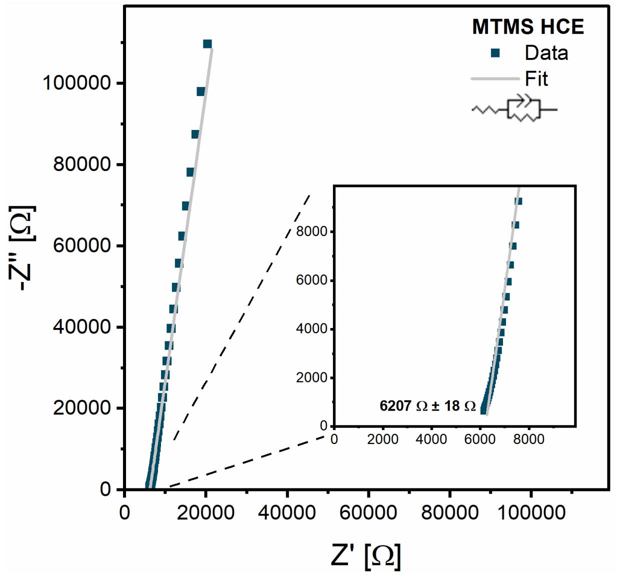

Figure S3: Nyquist plot of impedance data and equivalent circuit fit to determine the ionic conductivity. Note that the real and imaginary axes are not normalized to the area since the absolute resistance is required for calculation of the ionic conductivity.

The ionic conductivity (κ) is expressed as:

$$\kappa = \frac{L}{R_{el}A} \tag{1}$$

With L/A being defined as cell constant (C_c):

$$\kappa = \frac{C_c}{R_{el}} \tag{2}$$

For MTMS HCE (**Figure S3**) measured with cell and channel f4 (see **Table S3**):

$$\kappa = \frac{4.63}{6207}\ [S\ cm^{-1}] = 7.488\ 10^{-4}\ [S\ cm^{-1}]$$

Table S3: Overview of the cell constants for every ionic conductivity cell and its dedicated channel.

Cell	C_C [cm^{-1}]	Cell	C_C [cm^{-1}]	Cell	C_C [cm^{-1}]	Cell	C_C [cm^{-1}]
A1	0.00	D1	5.98	G1	4.74	L1	4.56
A2	4.59	D2	4.63	G2	4.63	L2	4.62
A3	4.75	D3	4.73	G3	4.70	L3	4.63
A4	4.58	D4	4.65	G4	4.76	L4	4.67
A5	4.72	D5	4.70	G5	4.65	L5	4.65
A6	4.72	D6	4.71	G6	4.55	L6	4.62
A7	4.67	D7	4.60	G7	4.70	L7	4.75
A8	4.70	D8	4.65	G8	-	L8	4.55
B1	4.70	E1	4.71	H1	-	M1	4.69
B2	4.68	E2	-	H2	-	M2	4.69
B3	4.71	E3	4.62	H3	-	M3	4.67
B4	4.74	E4	4.62	H4	-	M4	4.70
B5	4.69	E5	4.68	H5	-	M5	4.60
B6	4.63	E6	4.68	H6	-	M6	4.59
B7	4.69	E7	4.66	H7	-	M7	4.69
B8	4.71	E8	4.62	H8	-	M8	4.58
C1	4.59	F1	4.63	K1	4.63	N1	4.43
C2	4.75	F2	4.62	K2	4.57	N2	4.54
C3	4.68	F3	4.58	K3	4.62	N3	4.46
C4	4.64	F4	4.63	K4	4.79	N4	-
C5	4.66	F5	4.60	K5	4.61	N5	-
C6	4.61	F6	4.60	K6	4.57	N6	-
C7	4.58	F7	4.58	K7	4.67	N7	4.50
C8	4.66	F8	4.57	K8	4.60	N8	4.79

PFG NMR

All the NMR spectra were recorded on a BRUKER 4.7 T AVANCE III instrument using a commercially available BRUKER diff50 probe. Pulsed field gradient nuclear magnetic resonance (PFG-NMR) data was acquired with a (doubly tuned $^{7}Li/^{19}F$) 5 mm coil insert at (20.0 ± 0.1) °C. To avoid convection induced by temperature gradients, all the samples were equilibrated at the coil temperature for 30 min. To ensure reproducibility of the NMR self-diffusion measurements, ^{7}Li and ^{19}F standards were also measured at the start as well as the end of a measurement series, in this way accounting for any drift of the NMR system. The deviation of the ^{7}Li and ^{19}F diffusion coefficients from their literature value was always < ±2% before and after a measurement series. The maximum gradient strength, spread over 16 gradient steps, was optimized between 250 and 2947 G/cm for each sample. 8 scans per gradient step, a spoiler recovery of 2 s and a gradient pulse length δ of 1 ms were applied as the measurement parameters. The diffusion time Δ was kept at 20 ms for most of the samples. The corresponding ^{7}Li and ^{19}F NMR self-diffusion coefficients D were then derived from a stimulated echo sequence ("diffSte") after fitting the overall attenuated signal amplitudes (intensity) to the Stejskal-Tanner equation, which describes the case of rather ideal ("free") isotropic diffusion:

$$I = I_0 \times exp\left(-D\gamma^2\delta^2g^2\left(\Delta - \frac{\delta}{3}\right)\right) \quad (1)$$

In this case, I is the signal intensity at a given gradient strength, I_0 the initial signal in the absence of a magnetic field gradient and γ the gyromagnetic ratio. All the data analysis was performed with BRUKER Topspin 3.5.7 and BRUKER Dynamics Center 2.5.

Scanning electron microscopy

The *ex situ* morphology of lithium metal negative and NMC positive electrodes operated with DME- and MTMS-based MCE was investigated with a CrossBeam 550 scanning electron microscope (Carl-Zeiss). Cells were charged state to 4.3 V and subsequently disassembled in a glovebox (MBraun, O_2 < 0.5 ppm, H_2O < 0.5 ppm). Using a vacuum transfer module (Carl-Zeiss), positive and negative electrodes were transferred into the SEM without exposure to ambient conditions. Images were acquired using a field emission gun at a working distance of 4.9 mm and an accelerating voltage of 3 kV.

Table S4: Natural bond orbital analysis for the antibonding orbitals of tertiary atom in MTBE and MTMS, respectively.

	σ^* occupancy			
**Electrolyte Solvent**	***trans***	***cis***	***trans***	**Sum**
MTBE	0.035	0.023	0.035	0.094
MTMS	0.058	0.039	0.058	0.156

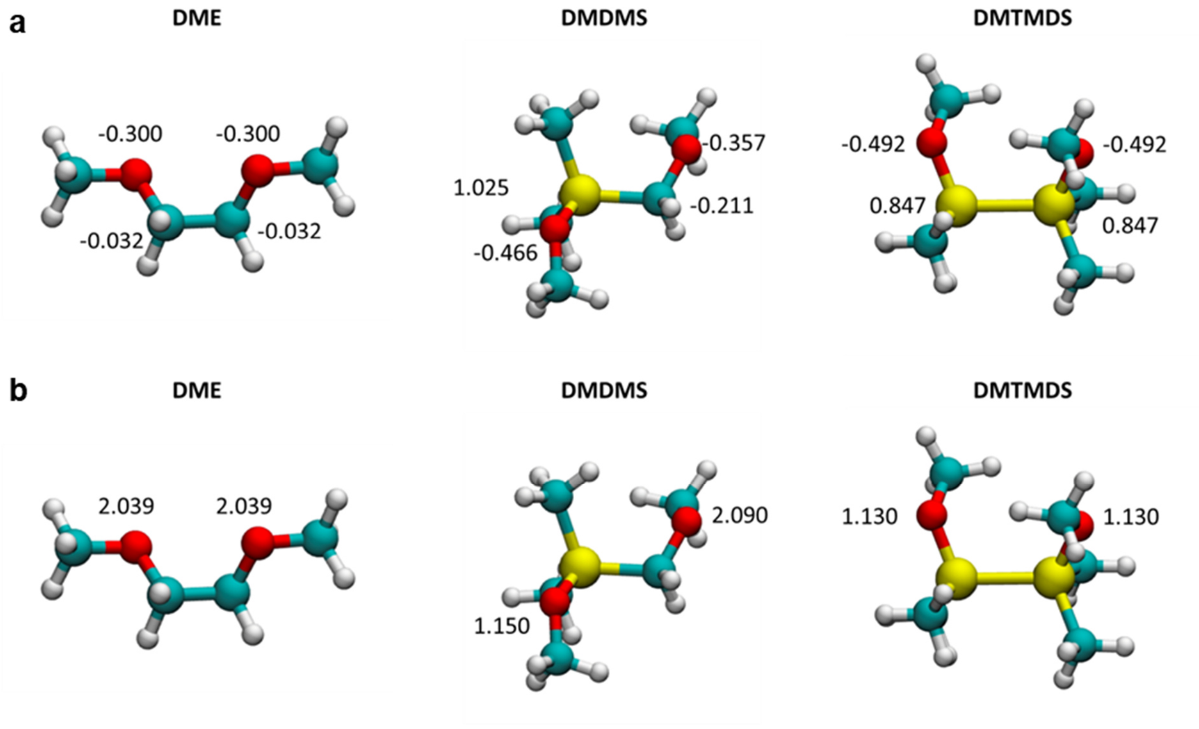

Figure S4: DFT-based analysis of (a) atomic charges and (b) population of electron density in the oxygen lone-pair orbital for DME and silicon substituted modifications thereof.

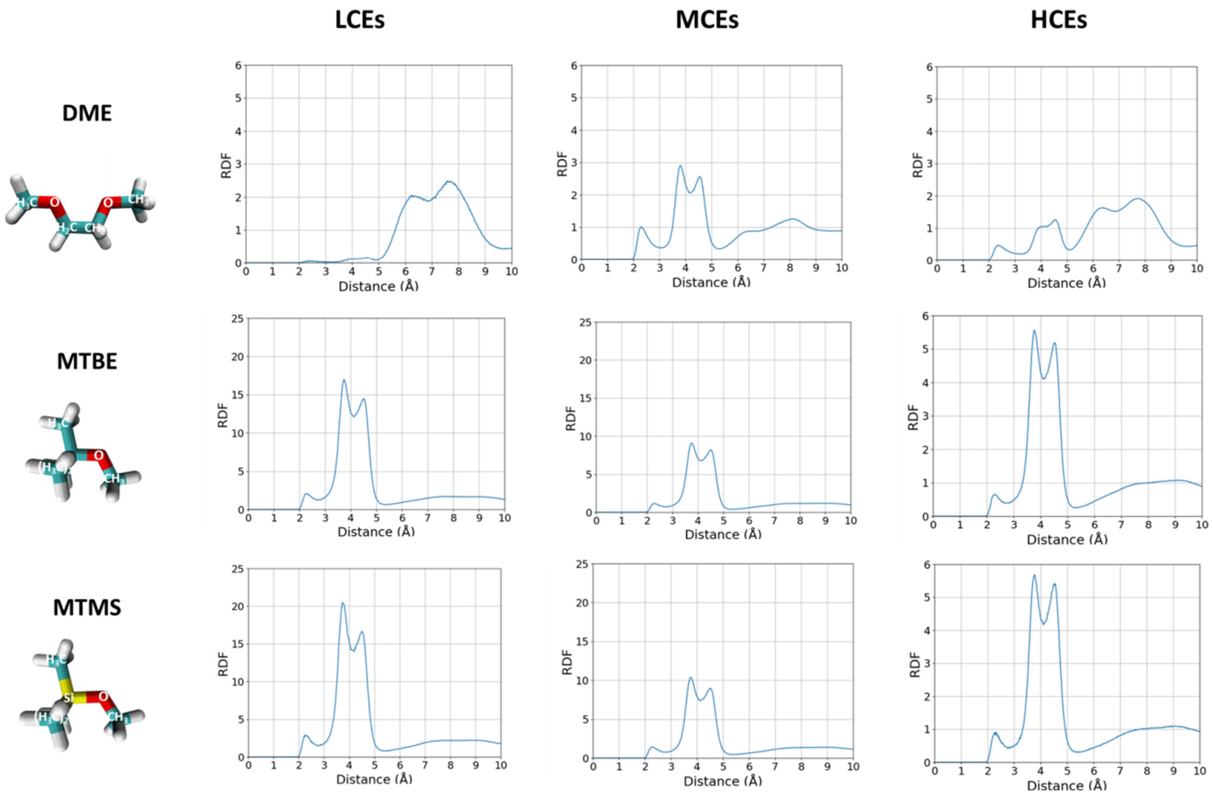

Figure S5: Radial distribution functions (RDFs) of nitrogen atoms around lithium ions in the different binary electrolyte formulations determined via molecular dynamic (MD) simulations. Note that differences in the intensity relate to the calculation of the RDFs, where density of the considered particles is related to the overall density of particles, scaling with the conducting salt to solvent ratio in this case.

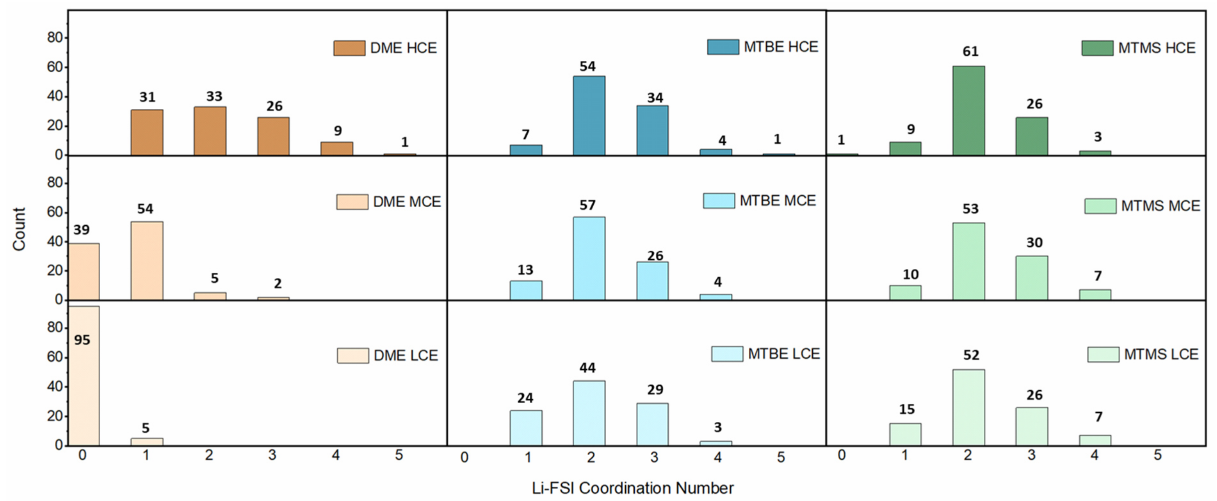

Figure S6: Histograms for the distribution of FSI^- anion coordination numbers in the considered electrolytes.

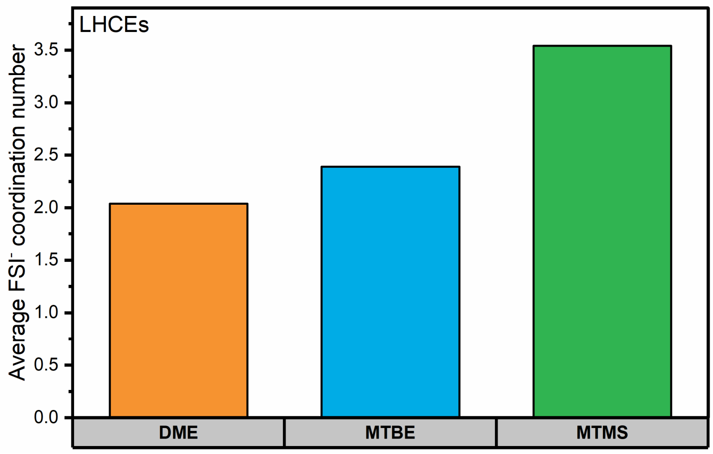

Figure S7: Average FSI- anion coordination numbers for localized high-concentration electrolytes containing DME, MTBE and MTMS determined by analyzing MD simulations.

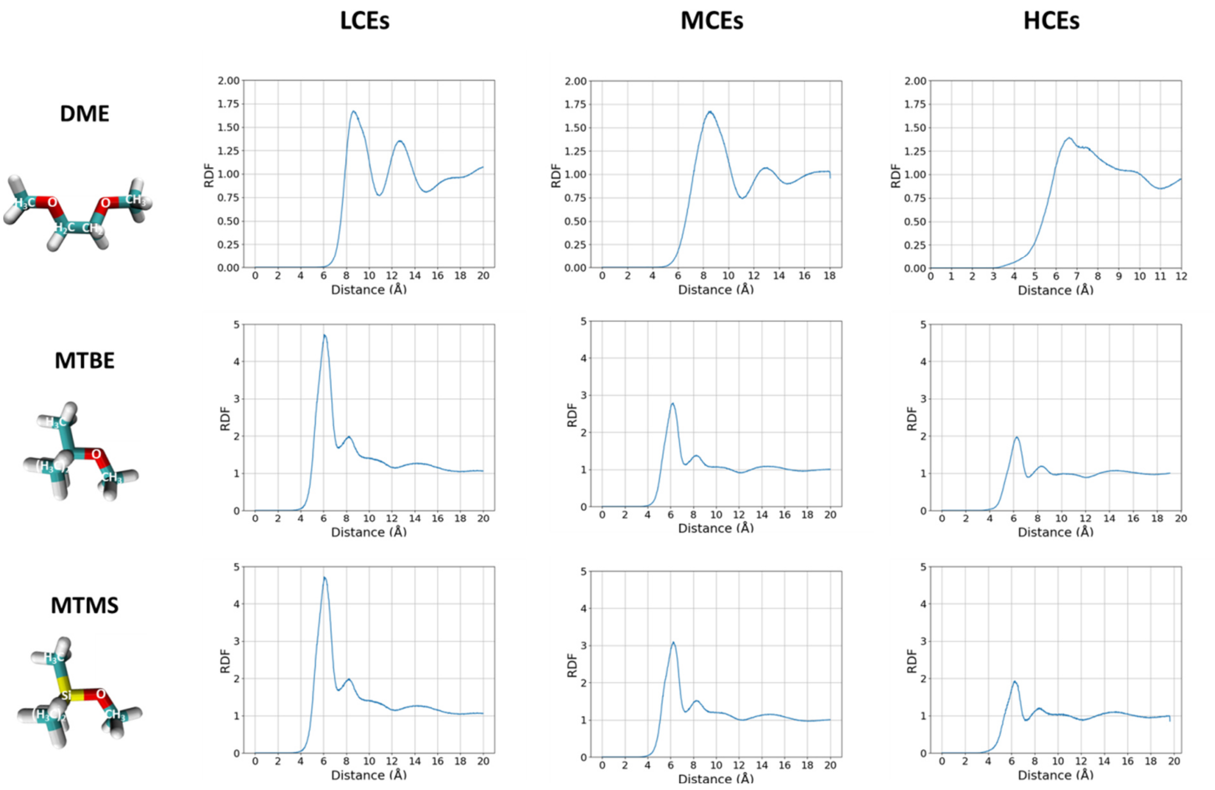

Figure S8: RDFs of lithium ions around lithium ions in the different binary electrolyte formulations determined via MD simulations. Since the radial density of the atoms is considered relative to the overall density of said atoms in the system, the intensity of peaks scale inversely with the salt to solvent ratio.

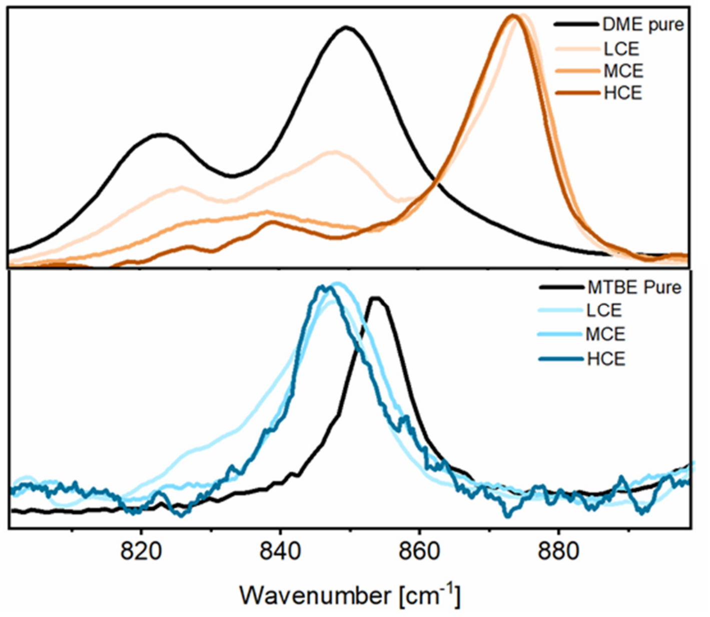

Figure S9: Comparison of solvent bands in Raman spectra for DME (O–C–C–O bands) and MTBE (O–C bands). All spectra are scaled to display comparable intensities.

Table S5: Comparison of experimentally and theoretically obtained Li^+ ion diffusion coefficients in the considered electrolytes.

Electrolyte		PFG-NMR D_{Li}^+ [m^2/s]	MD Simulation D_{Li}^+ [m^2/s]
LCEs	DME	1.50E-06	6.27E-07
	MTBE	8.09E-07	1.72E-06
	MTMS	4.92E-06	1.68E-06
MCEs	DME	3.41E-07	8.55E-08
	MTBE	1.12E-06	6.99E-07
	MTMS	2.31E-06	7.91E-07
HCEs	DME	1.44E-07	1.45E-08
	MTBE	2.36E-07	2.61E-08
	MTMS	3.54E-07	1.27E-07

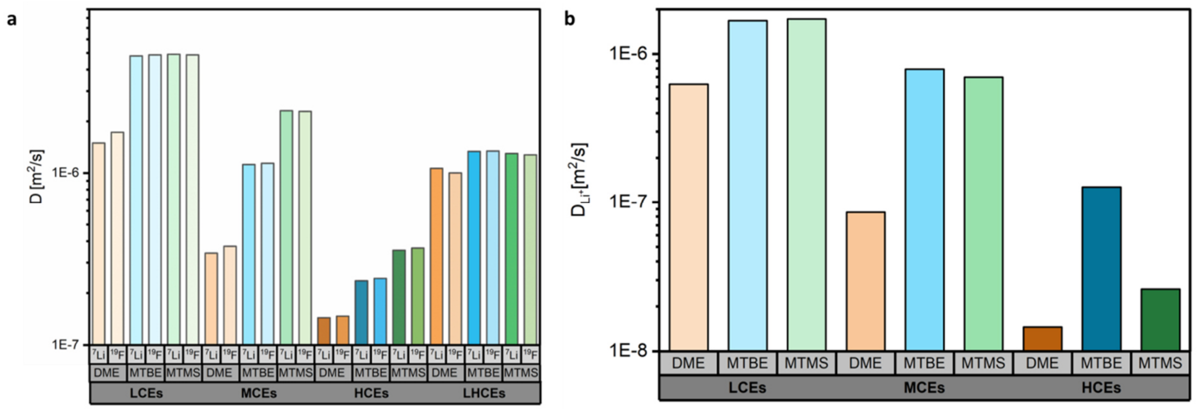

Figure S10: (a) Li^+ ion and FSI^- anion self-diffusion coefficients for DME-, MTBE- and MTMS-based electrolytes obtained from PFG-NMR spectroscopy and (b) FSI^- self-diffusion coefficients for DME-, MTBE- and MTMS-based electrolytes obtained from MD simulations.

Supplementary Note 1: A high entropy variant of MTMS-LHCE was prepared by mixing LiFSI, LiTFSI and MTMS in a molar ratio of 0.5 : 0.5 : 2.4, maintaining the molar ratio of conducting salt to solvent. The mixture was diluted with a 50:50 (wt:wt) mixture of TTE and BTFE (bis-2,2,2-trifluoroethyl ether). Here, the amount of diluent was maintained from the conventional MTMS LHCE (3 mol TTE), in order to achieve a similar conducting salt concentration and thus keep electrolyte compositions as comparable as possible. Despite significant improvements in ionic conductivities being reported,[27,28] HE-MTMS-LHCE displayed even slightly reduced ionic conductivity in our case (0.33 ± 0.06 mS/cm instead of 0.35 ± 0.01 mS/cm). Most likely, increased diversity of conducting salts, solvents or diluents is necessary to yield improvements in ionic conductivity. Since this is out of the scope of our study, no additional high entropy LHCEs were formulated and characterized.

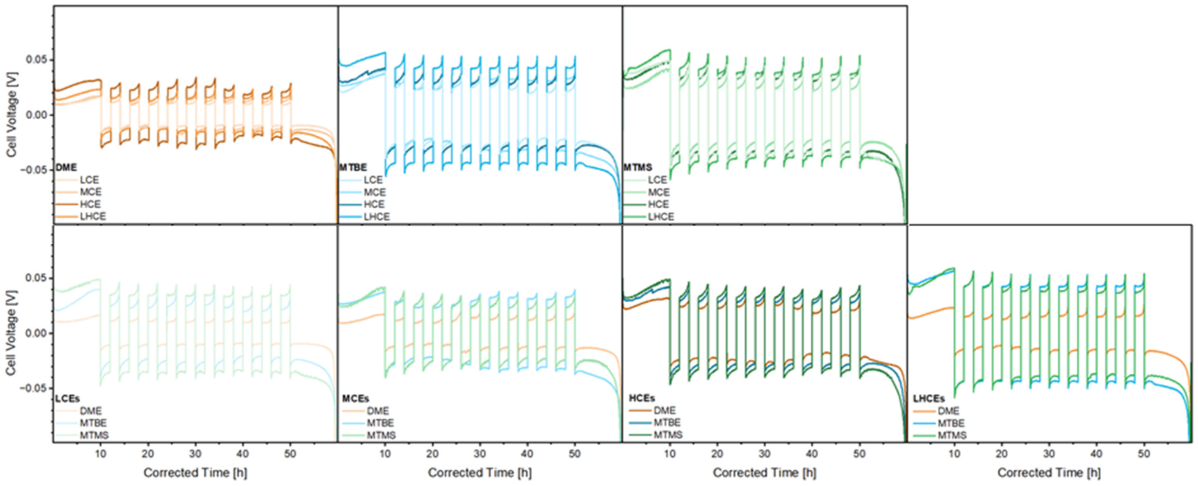

Figure S11: Comparison of voltage profiles for Li||Cu cells containing different electrolyte solvents and concentrations.

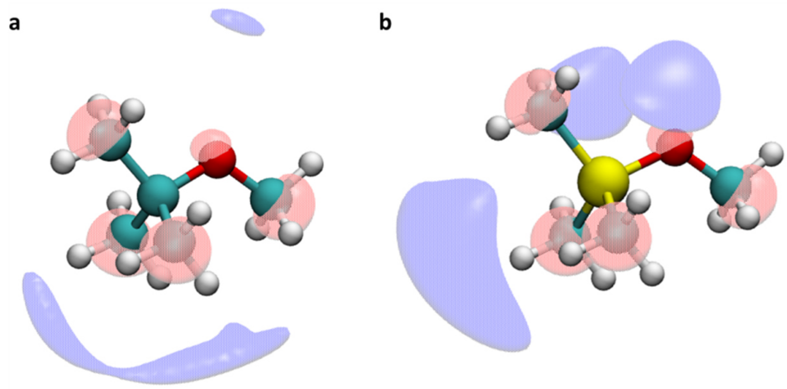

Figure S12: Spatial distribution of the LUMO for (a) MTBE and (b) MTMS.

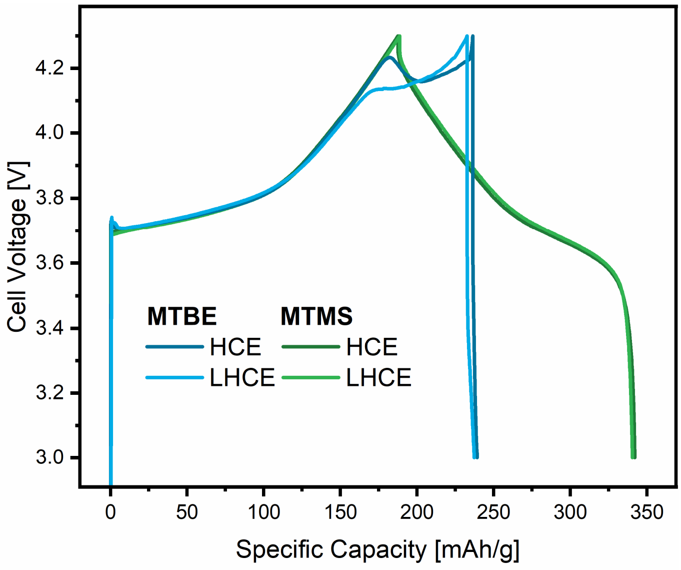

Figure S13: Voltage profiles for the 1st cycle of NMC622||Cu cells containing MTBE- and MTMS-based HCE and LHCE.

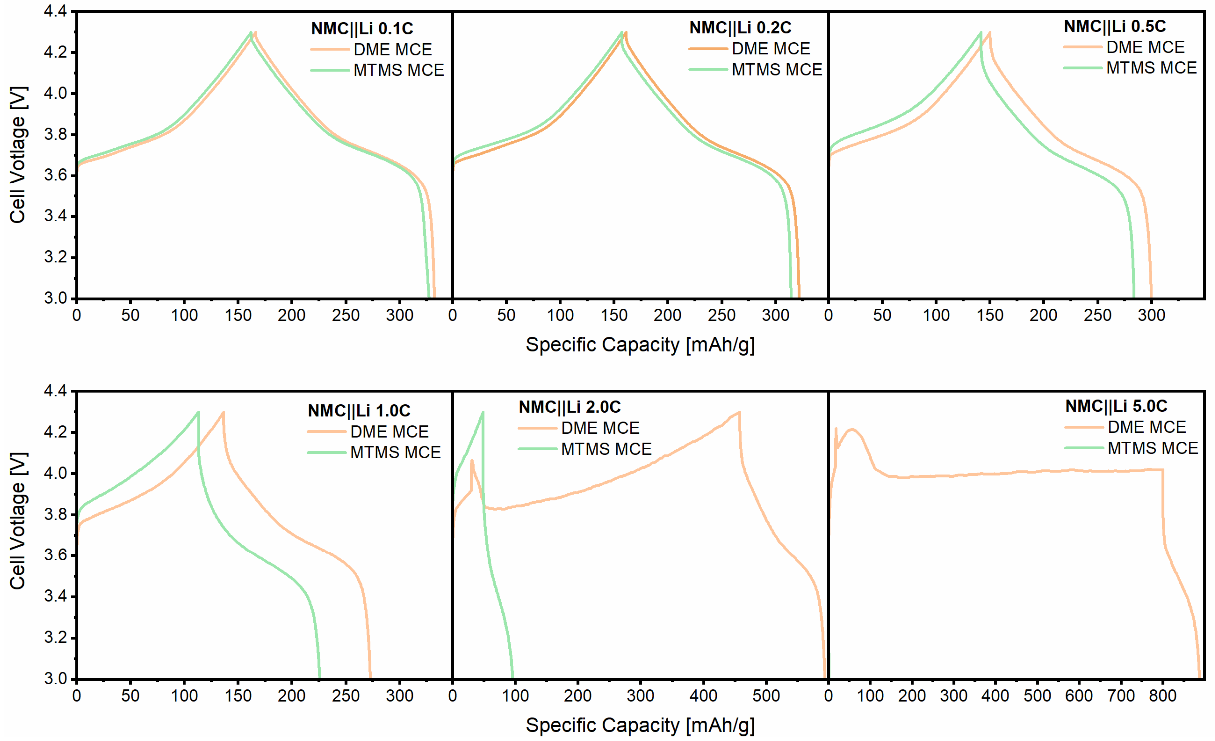

Figure S14: Voltage profiles of NMC622||Li cells operated with DME- and MTMS-based MCE at varying C-Rates.

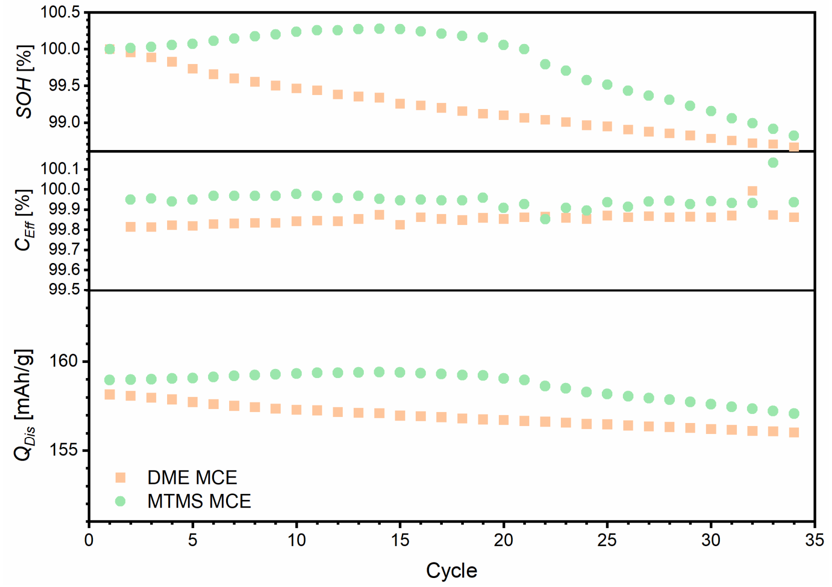

Figure S15: Electrochemical performance of NMC622||Li cells that were subjected to constant current operation at 0.2C (charge) and 0.3C (discharge) subsequent to the C-Rate test.

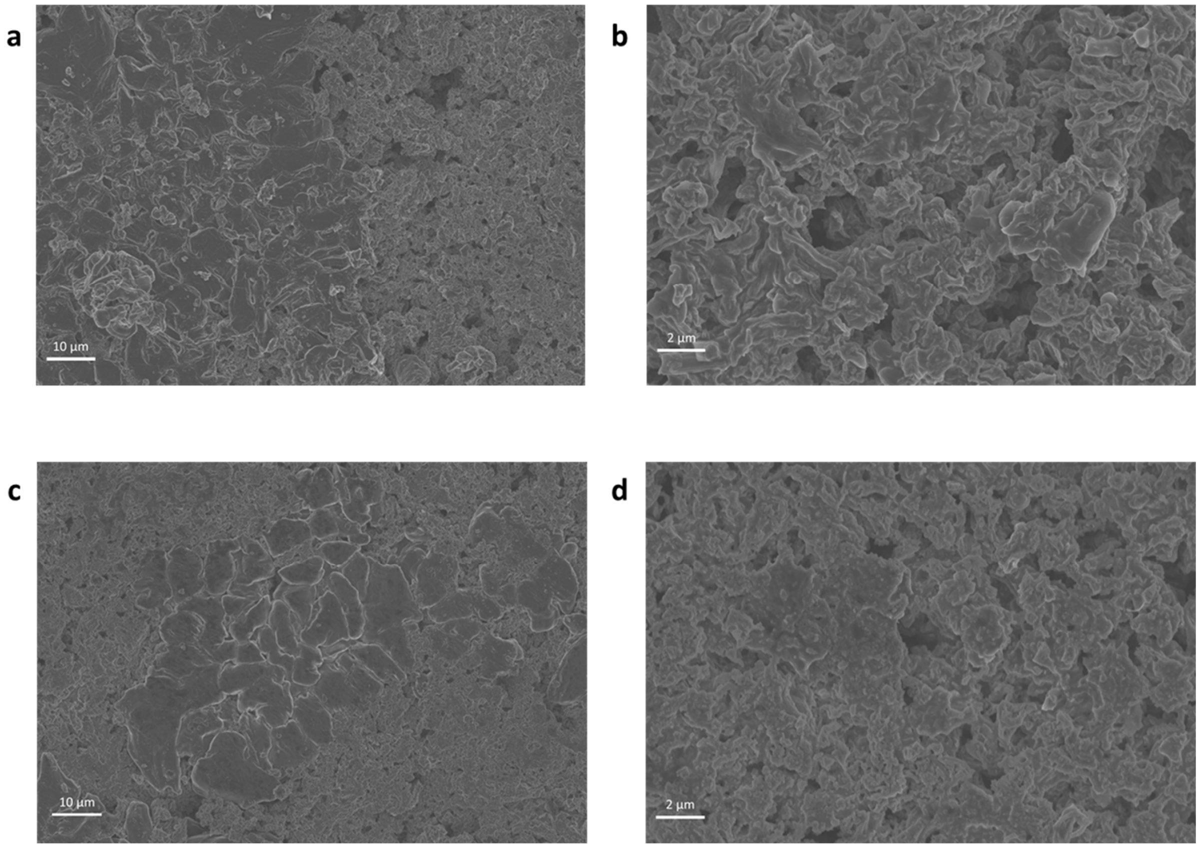

Figure S16: Scanning electron microscopy (SEM) images of the lithium metal negative electrodes from NMC622||Li coin cells with DME- and MTMS-based MCEs after completed lithium deposition (charge) in the 35th cycle following the C-Rate tests. (a) DME-based MCE, 1k magnification. (b) DME-based MCE, 5k magnification. (c) MTMS-based MCE, 1k magnification. (d) MTMS-based MCE, 5k magnification.

Synthesis of alternative electrolyte solvents

Besides the electrolyte solvents evaluated in the manuscript, other design strategies for weakly solvating electrolytes requiring synthesis of designed molecules were also explored. Approaches include increase of steric hindrance or decrease of Lewis basicity for coordinating units via substitution. Due to either the incapability of the designed electrolyte solvent to dissociate the conducting salt, enable a sufficient ionic conduction or instability upon electrochemical operation, these approaches were not discussed further in the manuscript.

1. General experimental

1.1. Glassware, Solvents and Reagents

All glassware and teflon-coated magnetic stir bars were dried in an oven at 80 °C prior to use. All anhydrous solvents were commercially supplied and stored over 3 Å mol. sieves or dried using an activated alumina column drying system (THF, MeOH). Reagents were purchased from commercial sources and used as received.

1.2. Chromatography and Data Analysis

Thin layer chromatography (TLC) was performed to monitor reactions when practical using Merck silica gel 60 F_{254} aluminum plates and visualized under UV light, or by staining with aqueous basic potassium permanganate followed by heating. **Flash column chromatography** (FCC) was carried out using Acros Organics silica gel (35–70 mesh) or a Biotage Isolera^{TM} flash purification system. **NMR spectra** were recorded on a Bruker Avance II 400, Agilent DD2 500 or DD2 600 spectrometers. All spectral data was acquired at 295 K. Deuterated solvents were purchased from Eurisotop ($CDCl_3$, deuteration > 99.8%). Chemical shifts (δ) are reported in parts per million (ppm) and referenced to $CDCl_3$ (^{1}H: 7.26 ppm; ^{13}C: 77.16 ppm). Coupling constants (J) are given in Hertz (Hz) and refer to corresponding multiplicities (s = singlet, d = doublet, t = triplet, q = quartet, quin = quintet, hex = hextet, h = heptet, m = multiplet, app = apparent, br. = broad signal, dd = doublet of doublets, etc.). The ^{1}H NMR spectra are reported as follows: chemical shift (multiplicity, coupling constants, number of protons). **High resolution mass spectra (HRMS)** were recorded using electrospray ionization (ESI) on a Bruker Daltonics, MicroToF spectrometer and calibrated using formate ion clusters.

1.3. Compound names

The names of compounds were generated by ChemDraw Professional 23.1 software (PerkinElmer), according to IUPAC nomenclature.

2. Experimental Data

1,1,2,2-Tetramethoxyethane

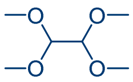

1

1,1,2,2-Tetramethoxyethane was synthesized via reported methods.[29] Afterwards the reaction mixture was purified via Kugelrohr distillation (110 °C, 60 mbar), yielding a colorless liquid (8.395 g, 55.90 mmol, 56%).

1,1,2,2-Tetramethoxyethane showed only low solubility of the conducting salt and thus exhibited insufficient conductivity for further electrochemical evaluation.

NMR Spectroscopy (*see spectra*):

^{1}**H NMR** (400 MHz, $CDCl_3$): δ_H 4.26 (s, 2H), 3.44 (s, 12H) ppm;

^{13}**C NMR** (101 MHz, $CDCl_3$): δ_C 103.5, 55.0 ppm.

HRMS (ESI^+): m/z calc'd for $C_6H_{14}O_4Na$ $[M+Na]^+$: 173.07843, found: 173.07845.

(2-Methoxyethyl)(methyl)sulfane

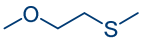

2

To an oven-dried 250 mL round-bottom flask equipped with a Teflon-coated magnetic stir bar was added 2-mercaptoethan-1-ol (2.09 mL, 2.34 g, 30.0 mmol, 1.00 equiv) in THF (75 mL, 0.4 M). After cooling the reactions mixture to 0 °C, sodium hydride (2.51 g, 63.0 mmol, 1.05 equiv) was added portionwise under strong stirring. The reaction mixture was stirred at 0 °C for 30 minutes. Iodomethane (3.92 mL, 8.94 g, 63.0 mmol, 1.05 equiv) was added dropwise at 0 °C and the reaction mixture was stirred at room temperature for 17 h. After this time, the solvent was carefully removed under reduced pressure and the crude product was purified by flash column chromatography on silica gel (98:2 pentane:Et_2O) to yield (2-Methoxyethyl)(methyl)sulfane as a colorless liquid (1.13 g, 10.7 mmol, 36%).

(2-Methoxyethyl)(methyl)sulfane showed sufficient solubility of the conducting salt. However, decomposition of the electrolyte solvent was observed in the voltage profile.

TLC: R_f = 0.70 (95:5 pentane:Et_2O).

NMR Spectroscopy (*see spectra*):

^{1}H NMR (400 MHz, $CDCl_3$): δ_H 3.56 (t, *J* = 6.7 Hz, 2H), 3.37 (s, 3H), 2.68 (t, *J* = 6.7 Hz, 2H), 2.14 (s, 3H) ppm;

^{13}C NMR (101 MHz, $CDCl_3$): δ_C 71.9, 58.8, 33.7, 16.1 ppm.

HRMS (ESI^+): m/z calc'd for $C_4H_{10}OSNa$ $[M+Na]^+$: 129.03446, found: 129.03441.

3. Spectroscopic Data

^{1}H NMR (400 MHz, $CDCl_3$) of 1,1,2,2-Tetramethoxyethane

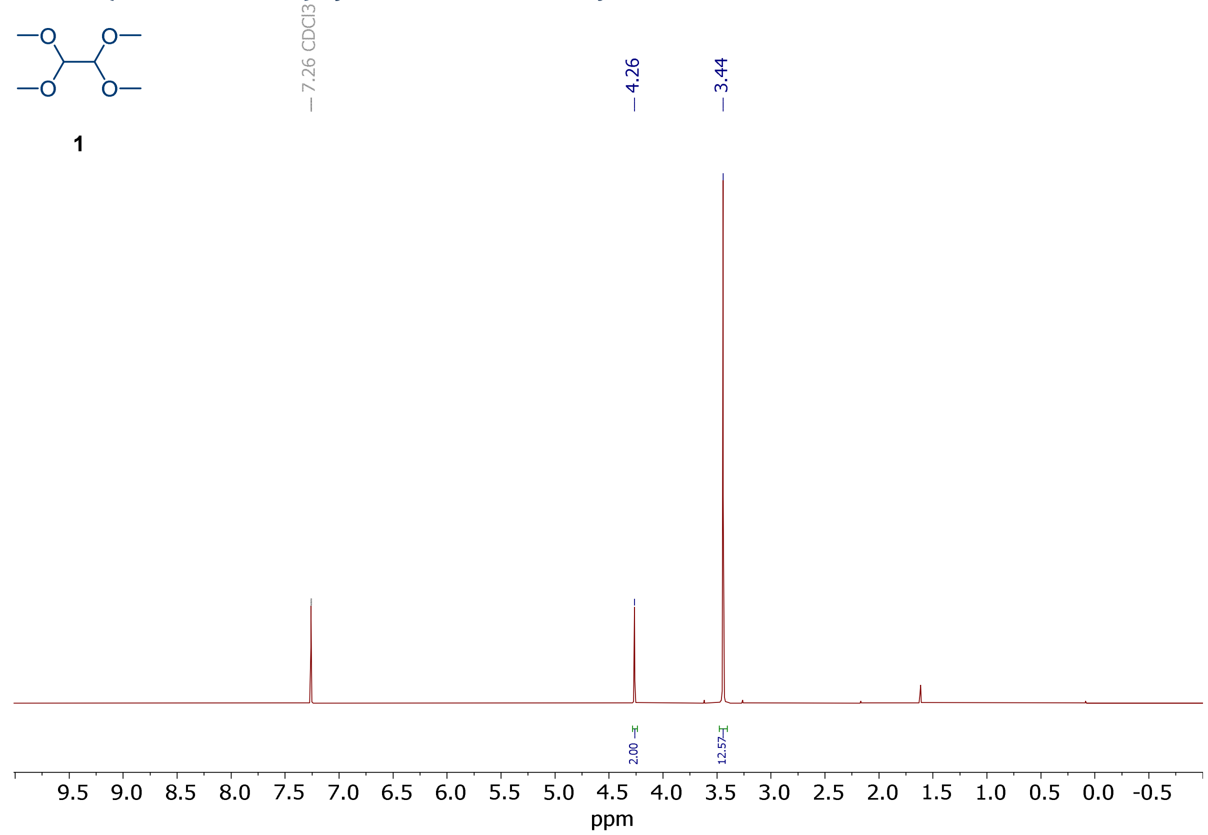

^{13}C NMR (101 MHz, $CDCl_3$) of 1,1,2,2-Tetramethoxyethane

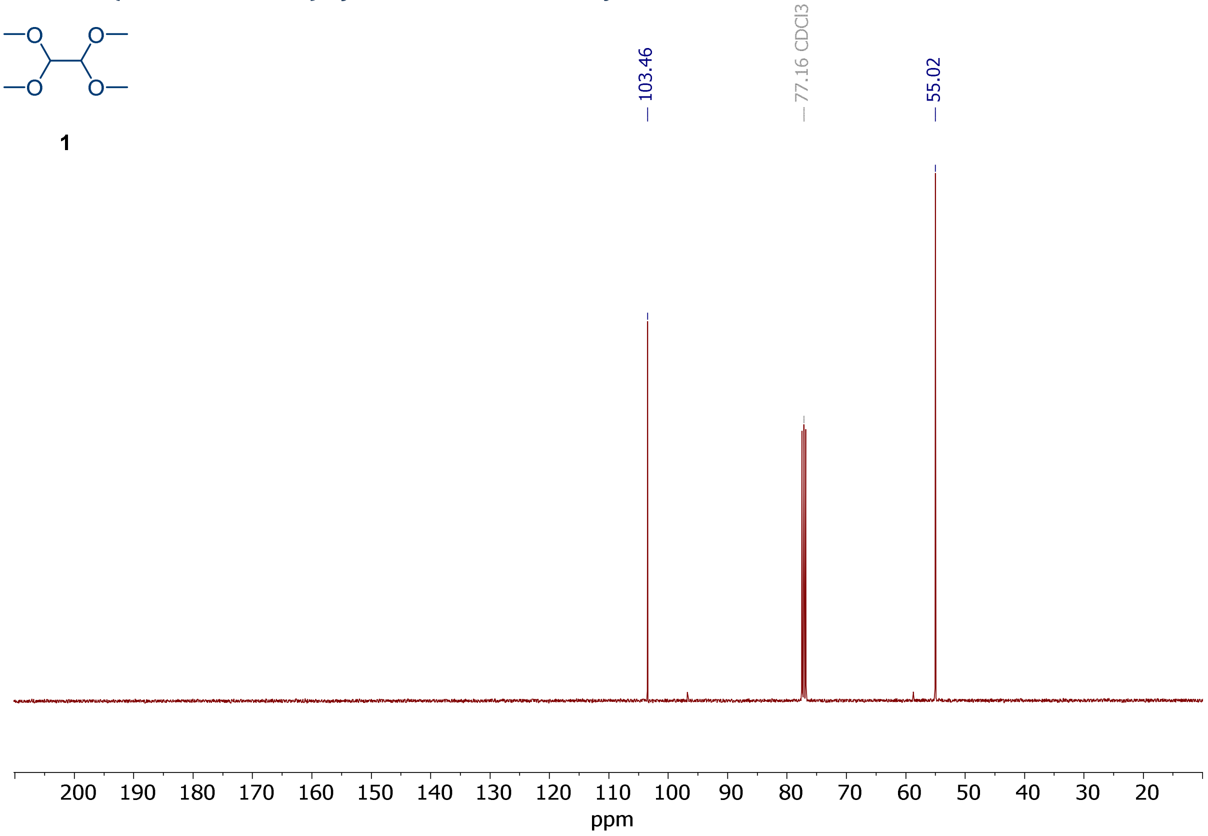

^{1}H NMR (400 MHz, $CDCl_3$) of (2-Methoxyethyl)(methyl)sulfane

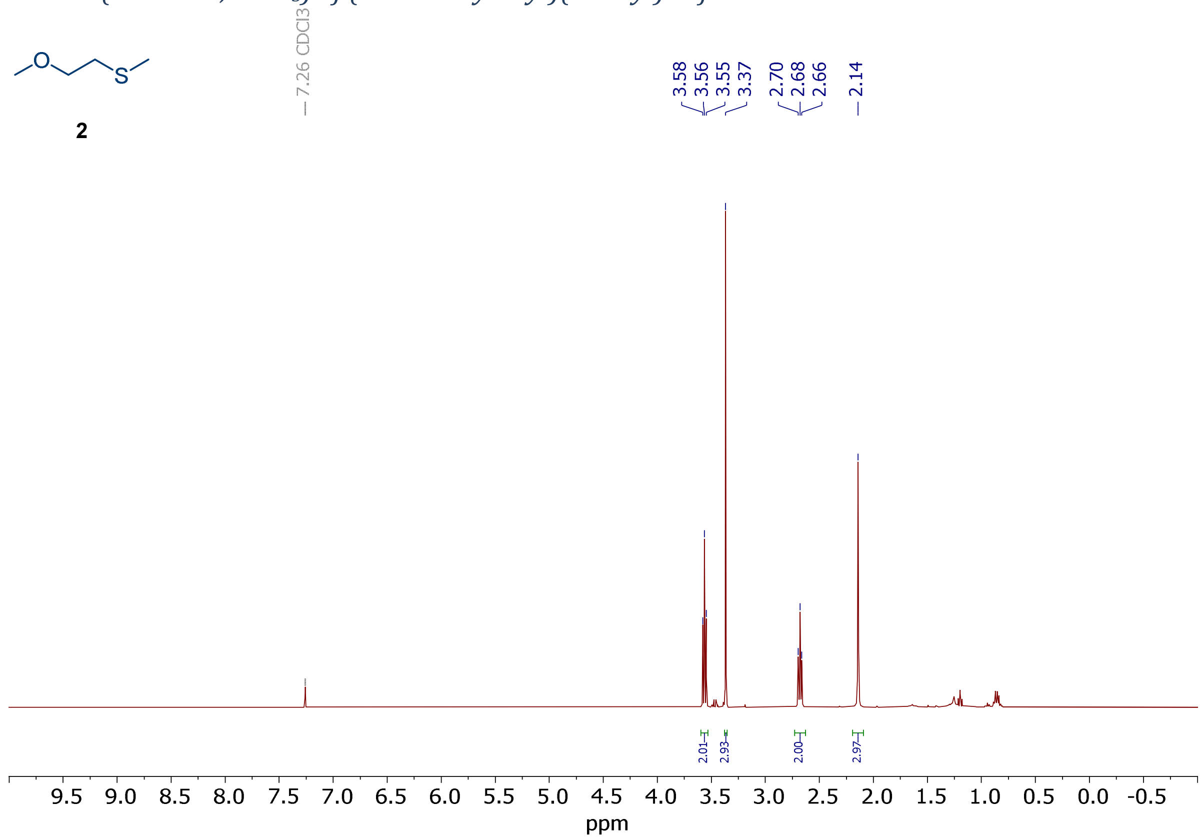

^{13}C NMR (101 MHz, $CDCl_3$) of (2-Methoxyethyl)(methyl)sulfane

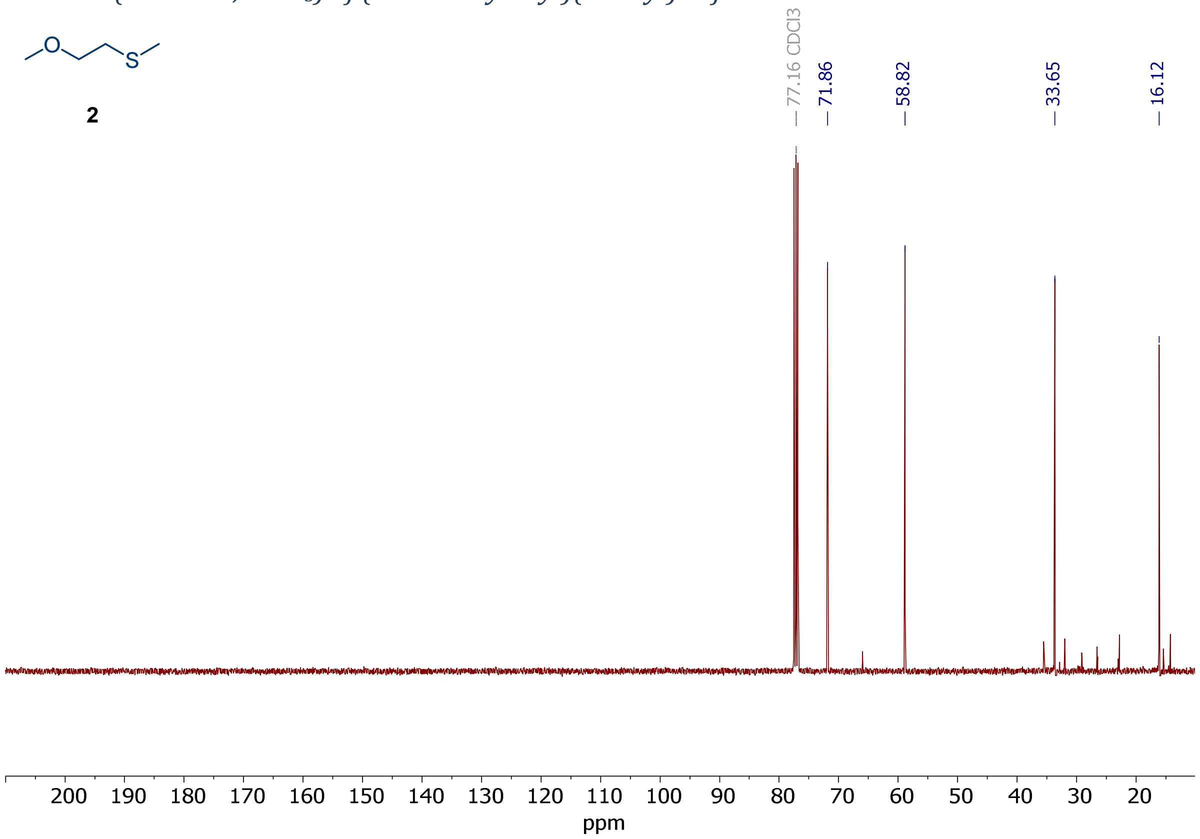

References

[1] N. Mardirossian, M. Head-Gordon, *The Journal of chemical physics* **2016**, *144*, 214110.
[2] F. Neese, *WIREs Comput Mol Sci* **2022**, *12*.
[3] F. Weigend, *Physical chemistry chemical physics : PCCP* **2006**, *8*, 1057.
[4] M. Garcia-Ratés, F. Neese, *J Comput Chem* **2020**, *41*, 922.
[5] C. M. Breneman, K. B. Wiberg, *J Comput Chem* **1990**, *11*, 361.
[6] J. Pipek, P. G. Mezey, *The Journal of chemical physics* **1989**, *90*, 4916.
[7] M. J. Frisch, G. W. Trucks, H. B. Schlegel, G. E. Scuseria, M. A. Robb, J. R. Cheeseman, G. Scalmani, V. Barone, G. A. Petersson, H. Nakatsuji et al., *Gaussian 16*, Gaussian Inc., Wallingford CT, **2016**.
[8] J.-D. Chai, M. Head-Gordon, *Physical chemistry chemical physics : PCCP* **2008**, *10*, 6615.
[9] R. A. Kendall, T. H. Dunning, R. J. Harrison, *The Journal of chemical physics* **1992**, *96*, 6796.
[10] T. H. Dunning, *The Journal of chemical physics* **1989**, *90*, 1007.
[11] E. D. Glendening, J. K. Badenhoop, J. K. Reed, J. E. Carpenter, J. A. Bohmann, C. M. Morales, C. R. Landis, F. Weinhold, *NBO 6.0*, Theoretical Chemistry Institute, Univerrsity of Wisconsin, Madison, WI, **2013**.
[12] M. Abraham, A. Alekseenko, V. Basov, C. Bergh, E. Briand, A. Brown, M. Doijade, G. Fiorin, S. Fleischmann, S. Gorelov et al., *GROMACS 2024.4 Manual*, Zenodo, **2024**.
[13] I. Leontyev, A. Stuchebrukhov, *Physical chemistry chemical physics : PCCP* **2011**, *13*, 2613.
[14] W. L. Jorgensen, D. S. Maxwell, J. Tirado-Rives, *J. Am. Chem. Soc.* **1996**, *118*, 11225.
[15] L. S. Dodda, I. Cabeza de Vaca, J. Tirado-Rives, W. L. Jorgensen, *Nucleic acids research* **2017**, *45*, W331-W336.
[16] J. N. Canongia Lopes, K. Shimizu, A. A. H. Pádua, Y. Umebayashi, S. Fukuda, K. Fujii, S. Ishiguro, *The journal of physical chemistry. B* **2008**, *112*, 9449.
[17] L. Martínez, R. Andrade, E. G. Birgin, J. M. Martínez, *J Comput Chem* **2009**, *30*, 2157.
[18] N. Michaud-Agrawal, E. J. Denning, T. B. Woolf, O. Beckstein, *J Comput Chem* **2011**, *32*, 2319.
[19] R. Gowers, M. Linke, J. Barnoud, T. Reddy, M. Melo, S. Seyler, J. Domański, D. Dotson, S. Buchoux, I. Kenney et al. in *Proceedings of the Python in Science Conference*, SciPy, **2016**, pp. 98–105.
[20] T. Beuse, M. Fingerle, C. Wagner, M. Winter, M. Börner, *Batteries* **2021**, *7*, 70.
[21] M. J. Lüther, S.-K. Jiang, M. A. Lange, J. Buchmann, A. Gómez Martín, R. Schmuch, T. Placke, B. J. Hwang, M. Winter, J. Kasnatscheew, *Small Structures* **2024**, *5*.
[22] D. Aurbach, Y. Gofer, J. Langzam, *J. Electrochem. Soc.* **1989**, *136*, 3198.
[23] B. D. Adams, J. Zheng, X. Ren, W. Xu, J.-G. Zhang, *Advanced Energy Materials* **2018**, *8*.
[24] A. Benayad, D. Diddens, A. Heuer, A. N. Krishnamoorthy, M. Maiti, F. Le Cras, M. Legallais, F. Rahmanian, Y. Shin, H. Stein et al., *Advanced Energy Materials* **2022**, *12*.
[25] A. Narayanan Krishnamoorthy, C. Wölke, D. Diddens, M. Maiti, Y. Mabrouk, P. Yan, M. Grünebaum, M. Winter, A. Heuer, I. Cekic-Laskovic, *Chemistry Methods* **2022**, *2*.
[26] A. Szczęsna-Chrzan, M. Vogler, P. Yan, G. Z. Żukowska, C. Wölke, A. Ostrowska, S. Szymańska, M. Marcinek, M. Winter, I. Cekic-Laskovic et al., *J. Mater. Chem. A* **2023**, *11*, 13483.
[27] Q. Wang, C. Zhao, J. Wang, Z. Yao, S. Wang, S. G. H. Kumar, S. Ganapathy, S. Eustace, X. Bai, B. Li et al., *Nature communications* **2023**, *14*, 440.
[28] Q. Wang, C. Zhao, Z. Yao, J. Wang, F. Wu, S. G. H. Kumar, S. Ganapathy, S. Eustace, X. Bai, B. Li et al., *Advanced materials (Deerfield Beach, Fla.)* **2023**, *35*, e2210677.
[29] F. H. Sangsari, F. Chastrette, M. Chastrette, *Synthetic Communications* **1988**, *18*, 1343.
